# Conserved HSP60 structure with lineage- and context-specific regulation in cnidarians

**DOI:** 10.26508/lsa.202503592

**Published:** 2026-06-24

**Authors:** Saborni Chowdhury, Avery J Kruger, Liza M Roger

**Affiliations:** 1 School of Molecular Sciences, Arizona State University, Tempe, AZ, USA; 2 School of Life Sciences, Arizona State University, Tempe, AZ, USA; 3 School of Earth and Space Exploration, Arizona State University, Tempe, AZ, USA; 4 School of Ocean Futures, Arizona State University, Tempe, AZ, USA

## Abstract

Heat shock proteins safeguard proteostasis under stress. We examined mitochondrial chaperonin HSP60 in three cnidarians to assess stress responses. We evaluated HSP60 expression in *Pocillopora acuta* (hard coral), *Exaiptasia diaphana* (sea anemone), and *Cassiopea xamachana* (upside-down jellyfish) using immunoblotting. In *P. acuta*, HSP60 was not detected at the fragment level under either control (25°C) or heat-stress (30°C). In contrast, isolated cells showed transient HSP60 expression under both temperature conditions, indicating context-dependent regulation in coral. *E. diaphana* and *C. xamachana* showed elevated HSP60 expression over 24 h when stressed (+5°C above laboratory optima). These patterns indicate lineage-specific regulatory mechanisms underlying chaperone-mediated stress response pathways. Thus, thermal sensitivity varies among species and across biological contexts. Consistent antibody cross-reactivity prompted evolutionary analysis. Phylogenetic analyses confirmed cnidarian HSP60 proteins are orthologous to vertebrate HSP60, demonstrating deep conservation across Metazoa. Although HSP60 is ancient and highly conserved, its role in regulating mitochondrial proteostasis varies across early-diverging metazoans. This study underlines the role of chaperone plasticity in cnidarian thermotolerance and diverging bleaching susceptibility of symbiotic cnidarians.

## Abstract (precis)

HSP60 regulation varies across three cnidarians (*P. acuta, E. diaphana, C. xamachana*). Cross-reactive antibodies and evolutionary analyses confirm orthology with human HSP60, highlighting lineage- and context-dependent regulation underlying cnidarian stress physiology and potential implications for coral bleaching under warming oceans.

## Introduction

Environmental stress responses vary widely among organisms, enabling some species to tolerate rapid environmental fluctuations, whereas others cannot (Schulte, 2014). Anthropogenic climate change, driven by fossil fuel combustion and habitat alteration, is amplifying major environmental stressors such as ocean warming, acidification, hypoxia, salinity shifts, and pollutant exposure (Killen et al, 2013; Kabir et al, 2023). These changes are disrupting marine ecosystems, impairing organismal physiology, and threatening the stability of oceanic food webs and ecosystem services (Killen et al, 2013; Hughes et al, 2018; Klein et al, 2018). Among the most vulnerable taxa are scleractinian corals and other cnidarians (e.g., sea anemones, jellyfish) whose symbioses underpin the biodiversity and productivity of coral reef ecosystems (Coker et al, 2014). Rising sea temperatures trigger the breakdown of symbiotic associations through the expulsion of the photosynthetic symbionts, leading to widespread bleaching and mortality (Baird et al, 2009).

Susceptibility to bleaching varies among symbiotic cnidarians. A central question in marine biology is how cnidarians and their symbiotic partners respond at the molecular level to thermal stress. One of the most conserved cellular defense systems involves heat shock proteins (HSPs), a family of ATP-dependent molecular chaperones that maintain proteostasis by refolding denatured proteins and preventing aggregation (Li & Srivastava, 2003; Mogk et al, 2019). Discovered by Ferruccio Ritossa in *Drosophila melanogaster* in the 1960s (Tissières et al, 1974; Welch, 1993), HSPs and the associated heat shock response represent one of the most ancient stress response pathways across all domains of life (Richter et al, 2010; Velichko et al, 2013). This conservation reflects strong evolutionary pressure to maintain cellular homeostasis under stress, with lineage-specific adaptations arising to meet distinct environmental challenges (Richter et al, 2010). Yet, despite this, the diversity and regulation of HSP expression in cnidarians remain poorly resolved, and we still lack a clear understanding of how the canonical heat shock response has been adapted to the distinct thermal niches and symbiotic lifestyles of these organisms.

Among the known HSP families, the major families (HSP60, HSP70, and HSP90, named according to their molecular weight) play a specialized role in stress mitigation. The canonical HSP90 acts as a constitutive stabilizer of signaling pathways, HSP70 is rapidly and highly inducible during acute stress, and HSP60 functions within mitochondria to refold imported or misfolded proteins under prolonged stress (Iwama et al, 1998; Kiang & Tsokos, 1998; Okamoto et al, 2017; Hess et al, 2018; Singh et al, 2024). In particular, HSP60 is the eukaryotic homolog of the bacterial chaperonin GroEL, suggesting evolutionary roots in prokaryotic proteostasis systems (Okamoto et al, 2017; Singh et al, 2024). Its structure and function are remarkably conserved between fruit flies, chickens, *E. coli*, and yeast (Richter et al, 2010; Okamoto et al, 2017; Hess et al, 2018; Singh et al, 2024), supporting a role as essential components of mitochondrial homeostasis (Iwama et al, 1998; Kiang & Tsokos, 1998). Whereas much is known about the conservation of this protein in particular in mammalian systems, much less is known about how this ancient chaperonin is regulated across evolutionarily distant yet functionally comparable lineages, especially in early-divergent animals such as cnidarians, where mitochondrial stress responses remain poorly characterized.

In marine invertebrates, HSP expression patterns are increasingly used as biomarkers of stress and resilience (Sørensen et al, 2003; de Jong et al, 2008; Ravindranathan et al, 2017). In stony corals, HSP expression and their corresponding encoding genes have been studied in relation to acclimation to elevated temperatures and carbon dioxide levels, whereas in anemones and jellyfish, HSP dynamics have provided insights into symbiotic flexibility and adaptive physiology (Pearl & Prodromou, 2001; Gunter & Degnan, 2007; Seveso et al, 2020; Vargas et al, 2022). The differential induction of heat shock response has also been reported in other cnidarians, such as the freshwater coelenterate *Hydra oligactis* (Bosch et al, 1988; Clark et al, 2008). Researchers compared protein expression in this hydra species with that of *Hydra attenuata* and found that *H. oligactis* polyps are extremely sensitive to thermal stress, suggesting that the classically described heat shock response is absent in the system (Bosch et al, 1988). Whereas the heat shock response has been identified in mammalian systems and many aquatic species, it seems to be absent from some systems such as *Hydra oligactis* and *Euplotes focardii* (Clark et al, 2008). Studies investigating comparative analyses linking the evolutionary conservation of HSP structure with the diversification of its regulatory behavior remain scarce. This disconnect limits our understanding of how conserved molecular components can yield divergent stress phenotypes across lineages.

Here, we first investigated the heat-responsive regulation of mitochondrial heat shock protein 60 (HSP60) across three ecologically and phylogenetically distinct symbiotic cnidarians: the scleractinian coral *Pocillopora acuta*; the model sea anemone *Exaiptasia diaphana*; and the upside-down jellyfish *Cassiopea xamachana*. These species differ in habitat, thermal tolerance, and the nature of their association with *Symbiodiniaceae* symbionts: *P. acuta* forms obligate mutualisms in reef environments, *E. diaphana* maintains facultative symbiosis, and *C. xamachana* perpetuates obligate symbiosis when inhabiting thermally variable mangroves and lagoons (Davy et al, 2012; Baumgarten et al, 2015; Fitt et al, 2021; Rivera Ortega & Thomé, 2023). By comparing their HSP60 expression under standardized heat-stress regimes, we aimed to characterize the conditions of chaperone regulation. Observed cross-reactivity of a mammalian HSP60 antibody across cnidarian taxa then motivated complementary phylogenetic analyses to assess the evolutionary conservation of this mitochondrial chaperone. Together, this integrative framework links physiological and evolutionary perspectives to explore how a deeply conserved molecular system operates across diverse cnidarian lineages.

## Results

### Organism level HSP60 protein expression across three cnidarians

**Figure 1.**
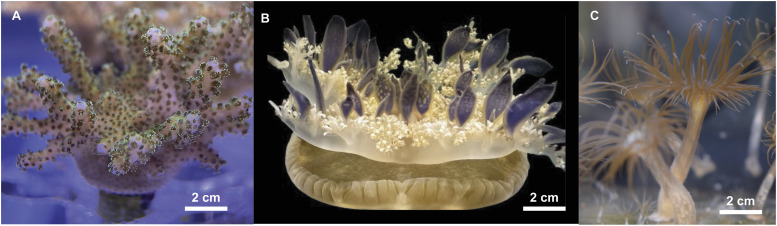
Photographs of symbiotic cnidarians used in this study. **(A)** *Pocillopora acuta* polyps (Scleractinia), a reef-building coral widely distributed across the Indo-Pacific. **(B)** *Cassiopea xamachana* medusa (Scyphozoa), upside-down jellyfish are native to the Caribbean and tropical western Atlantic. **(C)** *Exaiptasia diaphana* (Actiniaria), also known as the glass anemone, is predominantly found on warm-temperate to tropical shores of the Atlantic, Indo-Pacific, and Mediterranean.

**Figure 2.**
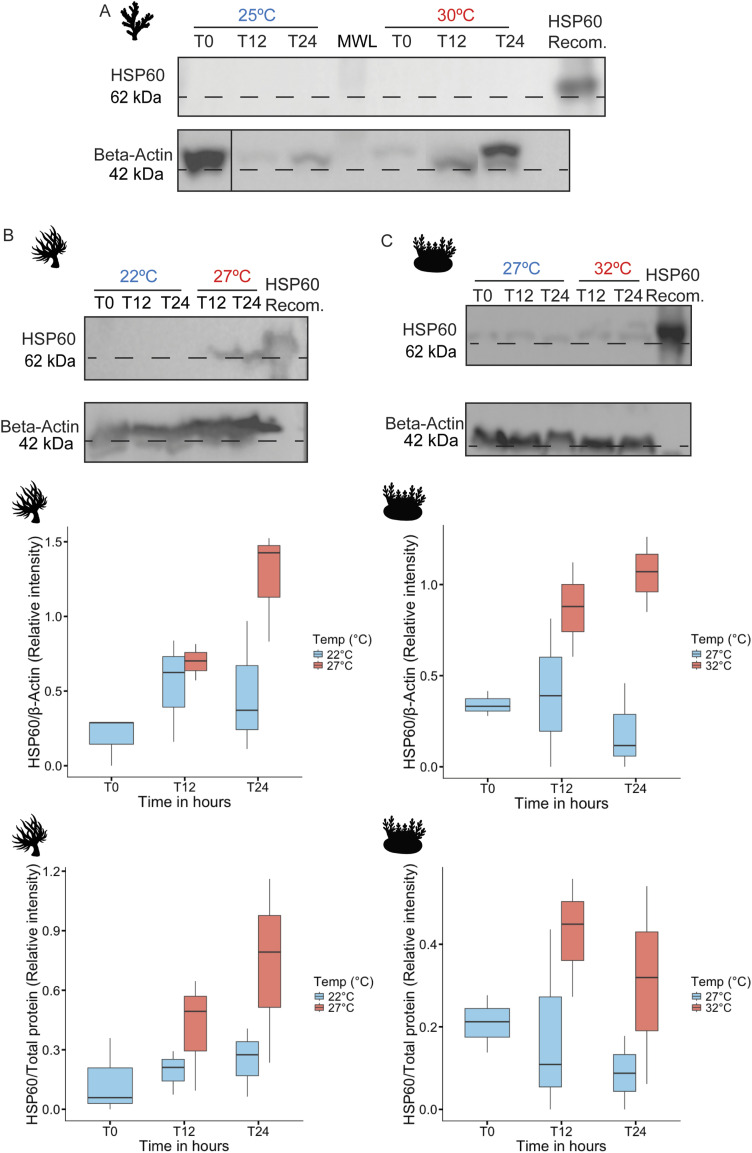
Quantitative Western Blot analysis of HSP60 across three species. **(A, B, C)** Immunoblot detection of HSP60 (∼62 kD) in tissue extracts at control (blue) and warm (red) temperatures and protein band density graphs (n = 3, ± SE) for *P. acuta* (A), *E. diaphana* (B), and *C. xamachana* (C). Samples were taken at various time points during 24 h exposure (T0, T12, T24). Recombinant HSP60 standard (Recom.) was included, and β-actin (∼42 kD) was used as a loading control. Both blots were run on the same gel. Western blot signals were quantified by densitometry and reported as relative band intensity, normalized to β-actin and total protein. Data presented as the mean of technical replicates for each biological replicate (n = 3, mean ± SE). Source data are available for this figure.

HSP60 induction was investigated across marine invertebrates belonging to three cnidarian classes (Fig 1A–C). Immunoblotting of whole-tissue lysates detected HSP60 across all three species (β-actin as protein loading control, protein band densitometry normalized to loading control), Fig 2. To account for variability in β-actin band intensity across lanes, we, in addition, normalized HSP60 band intensity to total protein. This analysis provided an independent framework for evaluating HSP60 expression patterns across species and experimental conditions. Relative HSP60 densitometry normalized to total protein is presented in Fig 2B and C.

In *P. acuta* tissue lysates, no detectable HSP60 signal was observed by western blot under the experimental conditions. In *P. acuta* fragments, HSP60 was not detected at either the control (25°C) or heated (30°C) conditions (Fig 2A). In *E. diaphana*, transient HSP60 expression was detected under control conditions (22°C) but gradually increased at 12 and 24 h under heat stress (27°C; Fig 2B). In *C. xamachana*, HSP60 expression was constitutively detected across all time points under both ambient (27°C) and heat stress (32°C) conditions (Fig 2C), with expression gradually increasing with exposure to elevated temperatures. Altogether, these data indicate species-specific basal abundance and heat inducibility of HSP60, with *P. acuta* showing no detectable response to heat stress of +5°C above its thermal optimum, *E. diaphana* showing temperature-responsive up-regulation, and *C. xamachana* exhibiting stable expression over the 24 h time course under control conditions and a slight temperature dependency under heated conditions. HSP60 was not detected in *P. acuta* tissue lysates under either control or heat stress conditions during the 24 h experimental period. However, during assay optimization under the same treatment conditions, a HSP60 band was observed in one heat-stressed tissue replicate, suggesting that HSP60 may be present at low or inconsistently detectable levels in *P. acuta* tissue (Table S1). Detection of recombinant HSP60 on the corresponding blots confirmed antibody reactivity under the assay conditions used. However, because recombinant HSP60 was not spiked into the cell lysates and a formal detection limit was not established for this matrix, we cannot fully exclude the possibility that low abundance or matrix-specific effects contributed to the lack of detectable signal in the sample. Accordingly, these findings are interpreted conservatively as variable or below detection-level expression rather than definitive absence. In addition to β-actin–based normalization, in *P. acuta* tissue, HSP60 was not detected under either control (25°C) or heat-stress (30°C) conditions (Fig 2A), (“not detected” defined as a signal below the detection threshold of the western blot assay). In *E. diaphana*, transient HSP60 expression was observed under control conditions (22°C) and increased at 12 and 24 h during heat stress (27°C; Fig 2B). In *C. xamachana*, HSP60 was detected constitutively across all time points under both ambient (27°C) and heat-stress (32°C) conditions (Fig 2C), with signal intensity increasing over time at elevated temperature. Whereas the representative blot image for *E. diaphana* (Fig 2B) is of lower visual quality, interpretation of this result is based on normalized densitometric measurements obtained from independent biological replicates rather than on visual assessment of the membrane image alone.

Table S1. Summary of HSP60 and β-actin detection across species, sample types, temperature treatments, and sampling time points.

### HSP60 expression under in vivo and in vitro heat stress in *P. acuta*

**Figure 3.**
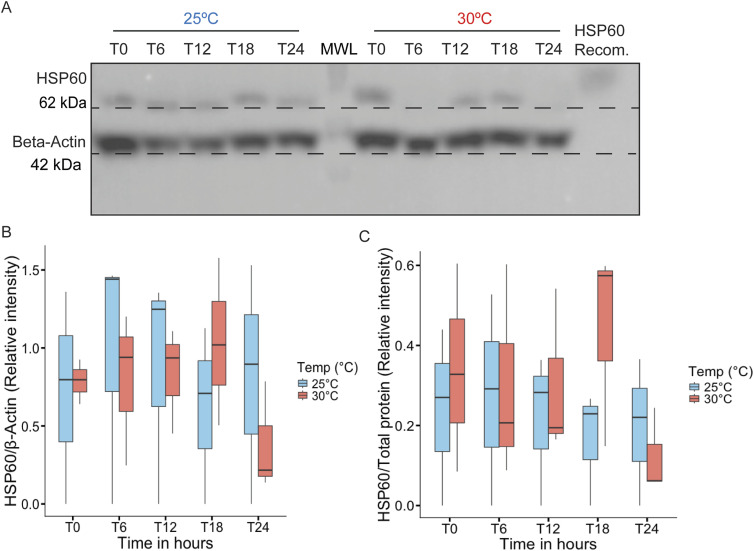
Western blot analysis of in vitro responses of *P. acuta* to acute heat stress. **(A)** Cellular-level stress assessed by HSP60 immunoblotting under control (blue) and heated (red) conditions and sampled at various time points over 24 h (T0—T24); (A) HSP60 band is visible at ∼62 kD, with β-actin (∼42 kD) as loading control. “Recom.” denotes the recombinant HSP60 standard, and MWL indicates the molecular-weight ladder. Representative Western blots and corresponding densitometric quantification of HSP60 under control and heat-stress conditions. **(B, C)** Densitometric measurements were performed in ImageJ after local background subtraction, and the HSP60 signal was normalized to the corresponding β-actin (B) and total-protein signal (C). Data are presented as relative band intensity and represent the mean of technical replicates for each biological replicate (n = 3, mean ± SE). Source data are available for this figure.

In contrast to the data presented above, in vitro assays of isolated *P. acuta* cells revealed variable HSP60 expression over exposure duration across replicates (Fig 3) when exposed to heat stress of +5°C above its thermal optimum (30°C) with a general temperature-dependent response and a basal but variable expression under control conditions (25°C; Fig 3A–C). These results indicate a context-dependent difference in HSP60 regulation in *P. acuta*, where isolated cells exhibit a transient, time- and temperature-dependent increase in HSP60 expression under heat stress, whereas intact tissue fragments do not show detectable induction under the same conditions.

### Statistical analysis of the two-way ANOVA test of HSP60 across cnidarian systems

Each analysis was performed on independent biological replicates (n = 3 per condition), and sample sizes differed across datasets depending on the number of time points included.

In *E. diaphana*, two-way ANOVA of β-actin-normalized HSP60 intensity revealed significant main effects of temperature (*F*_1,8_ = 5.405, *P* = 0.0486) and time (*F*_1,8_ = 5.721, *P* = 0.0437), whereas the temperature × time interaction was not significant (*F*_1,8_ = 0.844, *P* = 0.3852). In contrast, total-protein-normalized HSP60 intensity did not show significant effects of temperature (*F*_1,8_ = 4.311, *P* = 0.0715), time (*F*_1,8_ = 1.232, *P* = 0.2992), or their interaction (*F*_1,8_ = 0.606, *P* = 0.4588). Together, these results suggest a temperature-associated pattern in *E. diaphana*, with stronger statistical support under β-actin normalization than under total-protein normalization (Table S2).

Table S2. Statistical comparison of normalized HSP60 expression across cnidarian systems under control and heat-stress conditions.

In *C. xamachana*, β-actin-normalized HSP60 intensity showed a significant effect of temperature (*F*_1,8_ = 16.129, *P* = 0.00386), whereas time (*F*_1,8_ = 0.003, *P* = 0.96131) and the temperature × time interaction (*F*_1,8_ = 1.456, *P* = 0.26200) were not significant. Under total-protein normalization, the temperature effect did not reach significance but approached it (*F*_1,8_ = 4.685, *P* = 0.0623), whereas time (*F*_1,8_ = 0.985, *P* = 0.3501) and the interaction (*F*_1,8_ = 0.015, *P* = 0.9055) remained non-significant. Results suggest that HSP60 expression in *C. xamachana* was more strongly associated with temperature than with exposure duration during the sampled period (Table S2).

In dissociated *P. acuta* cells, two-way ANOVA did not detect significant effects of temperature (*F*_1,20_ = 0.019, *P* = 0.893), time (*F*_4,20_ = 0.228, *P* = 0.920), or their interaction (F4,20 = 0.428, *P* = 0.787) when HSP60 was normalized to β-actin. The same pattern was observed with total-protein normalization, where temperature (*F*_1,20_ = 1.103, *P* = 0.306), time (*F*_4,20_ = 0.433, *P* = 0.783), and the temperature × time interaction (*F*_4,20_ = 0.519, *P* = 0.723) were all non-significant. Thus, although HSP60 signal was detectable in dissociated *P. acuta* cells, it was not significantly regulated across the tested conditions (Table S2).

For the datasets analyzed by ANOVA, inspection of residuals, along with Shapiro–Wilk and Levene’s tests, did not indicate major violations of model assumptions.

### Multiple sequence alignment reveals high conservation of functional motifs

**Figure 4.**
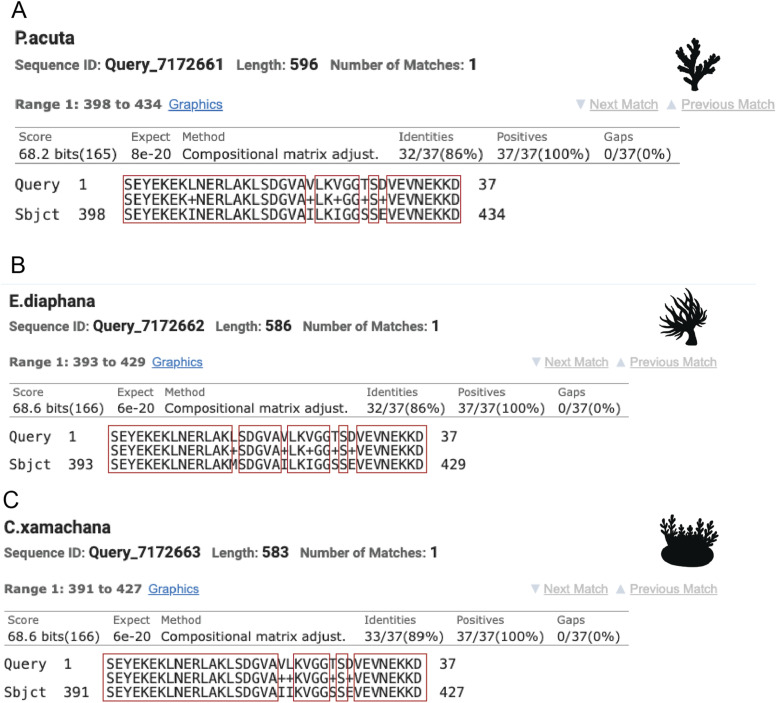
Conserved HSP60 motif shared across Cnidaria and humans. **(A, B, C)** Pairwise BLASTp alignments of human mitochondrial HSP60 (HSPD1) with HSP60 orthologs from (A) *P. acuta* (596 aa), (B) *E. diaphana* (586 aa), and (C) *C. xamachana* (583 aa) reveal a highly conserved 37 aa segment within the C-terminal region (alignment ranges: ∼391–434). Identities are 86% (*P. acuta* and *E. diaphana*) and 89% (*C. xamachana*), with 100% positives and no gaps (E-values between 8 × 10^−20^ and 6 × 10^−20^).

The candidate HSP60 orthologs were identified in *P. acuta* (ID: TCONS_00030188), *E. diaphana* (ID: AIPGENE15267 [P18687]), and *C. xamachana* (ID: Casxa1|9735) through BLASTp searches against the canonical human HSPD1 sequence (UniProt ID: P10809). All three cnidarian proteins exhibited strong homology to the mitochondrial chaperonin 60 family (query coverage ≥80%), confirming their identity as HSP60 orthologs. Multiple sequence alignments (MSAs) between the cnidarian and human HSP60 proteins revealed extensive conservation across key functional motifs, with over 85% sequence identity across the aligned regions (Fig 4A–C, Table S3), indicating high structural and functional similarity. Furthermore, sequence comparison across the full-length protein demonstrated overall conservation ranging from 69.6% to 91% identity and 84–96% positives relative to human HSPD1 (Table S3).

Table S3. Table showing alignment of candidate sequences.

**Figure S1.**
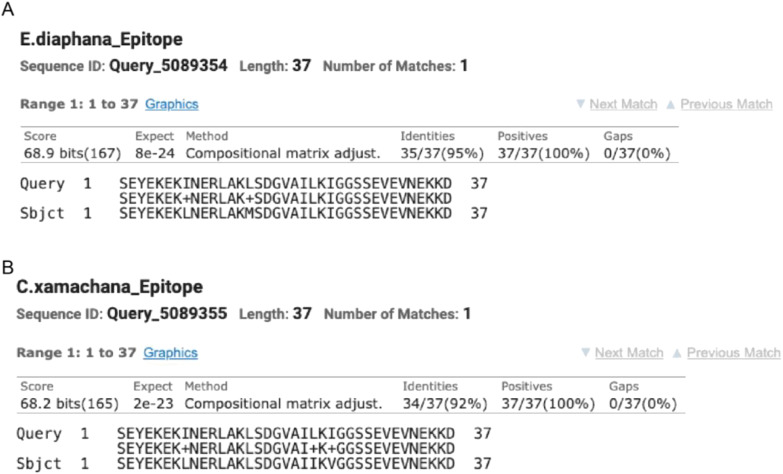
Conservation of C-terminus HSP60 antigenic epitope across study species. **(A, B)** Pairwise BLASTp alignments using a 37 amino acid epitope from *P. acuta* HSP60 (query) show strong conservation in (A) *E. diaphana* (35/37 identities; 95% identity; 100% positives; 0 gaps; E-value 8 × 10^−24^) and (B) *C. xamachana* (34/37 identities; 92% identity; 100% positives; 0 gaps; E-value 2 × 10^−23^). The high sequence identity across Anthozoa and Medusozoa supports cross-reactivity of anti-HSP60 antibodies and highlights broad phylogenetic conservation of this epitope among cnidarians.

Epitope analysis of the experimentally validated human HSP60 antibody region (aa 383–419, Thermo Fisher Scientific) showed partial to complete conservation across the corresponding cnidarian residues (Fig S1A and B, Table S4). Predicted epitope identity scores (86% for *P. acuta* and *E. diaphana*, 89% for *C. xamachana*) further support strong antibody cross-reactivity potential and underscore the deep conservation of antigenic and functional domains within the HSP60 family.

Table S4. Table showing epitope alignments among candidate sequences.

### A structurally conserved HSP60 epitope spans cnidaria and mammalia

**Figure 5.**
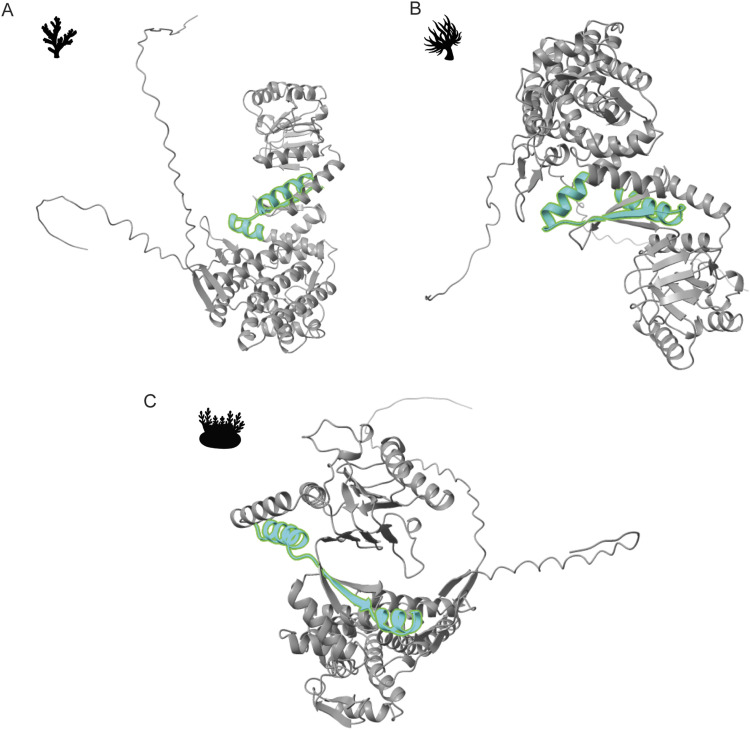
AlphaFold-predicted tertiary structures of HSP60 homologs in three cnidarian species. **(A, B, C)** Monomeric HSP60 models for (A) *P. acuta*, (B) *E. diaphana*, and (C) *C. xamachana* generated using AlphaFold3. The conserved 37-residue epitopes (green) identified by pairwise alignments are highlighted in each model and map to an equivalent region within the equatorial domain. Per-residue confidence (pLDDT) and predicted aligned error are consistent with high confidence in the globular cores and modestly lower confidence at hinge/loop regions (pTM = 0.73 for *P. acuta*, pTM = 0.76 for *E. diaphana* and pTM = 0.78 for *C. xamachana*). Canonical HSP60 functions in vivo as a tetradecameric double ring; only the monomeric fold is shown here for clarity.

To connect sequence conservation to structural context, we predicted HSP60 tertiary structures for *P. acuta*, *E. diaphana*, and *C. xamachana* using AlphaFold3 and mapped the 37-residue antigenic motif identified by pairwise alignments to human HSP60 (HSPD1, Fig 5A–C) (Jumper et al, 2021). AlphaFold3 consistently recovered the canonical domain organization across all three cnidarians, placing the conserved epitope in an equivalent position on the equatorial domain surface (Fig 5A–C). This structural correspondence provides a clear rationale for the observed antibody cross-reactivity and indicates strong evolutionary constraint on this region. The equatorial domain mediates ATP binding and oligomerization in Group I chaperonins (Clare et al, 2012; Vilasi et al, 2018), and the conservation of its surface-exposed epitope across taxa supports an essential mechanistic role. All predicted cnidarian HSP60 models display the canonical architecture with an extended C-terminal tail (Fig 5A–C), within which the conserved motif remains solvent exposed on the monomer surface. The shared positioning and accessibility of this epitope among cnidarians and mammalian homologs highlight deep functional conservation of the HSP60 complex across Metazoa.

### Phylogenetic analysis supports evolutionary conservation across cnidaria

**Figure S2.**
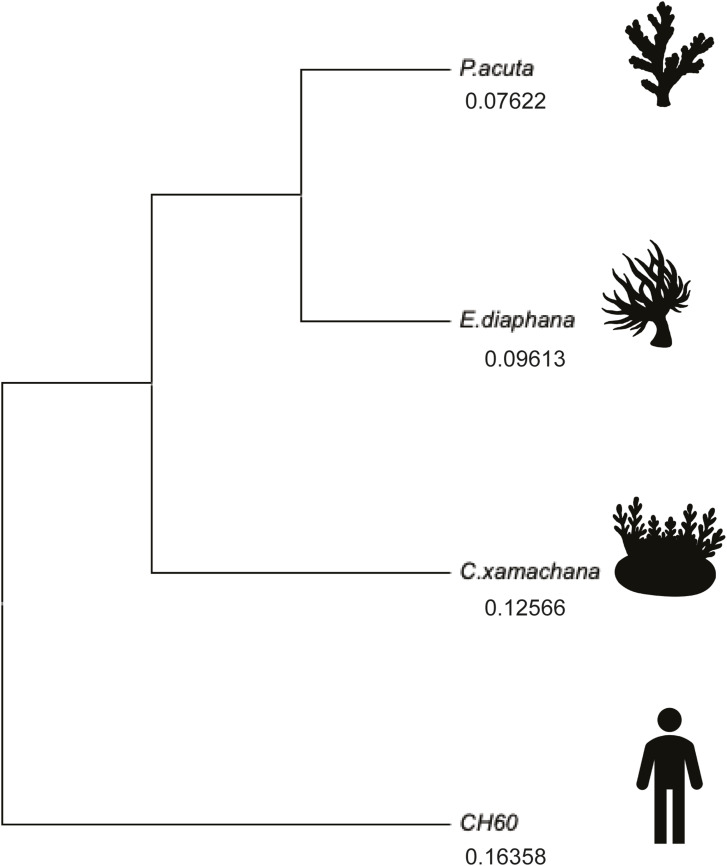
Phylogenetic placement of cnidarian HSP60 proteins. Rooted tree based on HSP60 (chaperonin 60) protein sequences from *P. acuta* (stony coral), *E. diaphana* (sea anemone), and *C. xamachana* (the upside-down jellyfish), with human HSP60/HSPD1 (CH60) used as the outgroup.

Maximum-likelihood phylogenetic analysis of aligned HSP60 sequences recovered the expected relationships among the three cnidarians (Fig S2). The coral *P. acuta* (Scleractinia) and the sea anemone *E. diaphana* (Actiniaria) formed a well-supported sister clade, consistent with the established Anthozoa phylogeny (Zapata et al, 2015). The jellyfish *C. xamachana* branched separately, reflecting its placement within the medusozoan lineage. The tree was rooted with the human mitochondrial HSP60 (HSPD1/CH60), which served as an outgroup to Cnidaria. This topology confirms that cnidarian HSP60 proteins are orthologous to the human homolog and that the locus retains sufficient phylogenetic signal to resolve divergence within Cnidaria.

### TargetP-2.0 analysis confirms mitochondrial localization of candidate HSP60 sequences

To verify subcellular targeting of the cnidarian HSP60 candidates, we used the TargetP-2.0 prediction tool, which estimates the likelihood of a protein being mitochondrial in origin. Given that HSP60 is a mitochondrial chaperonin in mammals, this analysis was performed to confirm whether the cnidarian homologs share the same localization.

**Figure S3.**
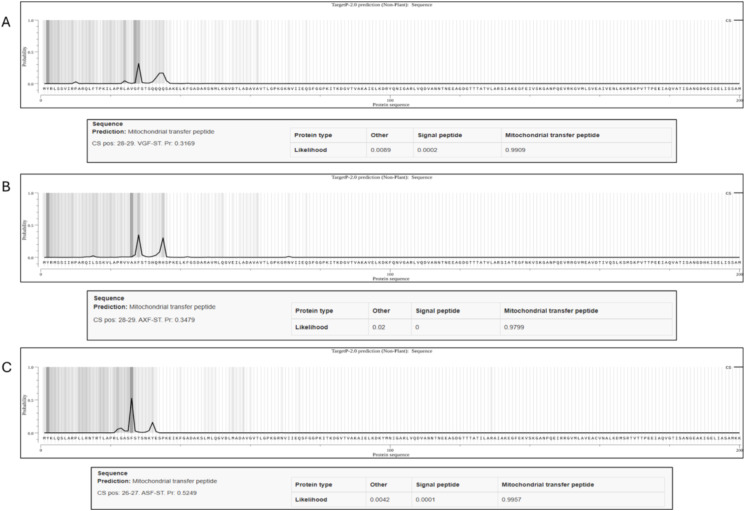
TargetP-2.0 outputs confirming mitochondrial localization. **(A, B, C)** Generated graphs confirming the location and confirmation of candidate sequences for mitochondrial HSP60 in (A) *P. acuta*, (B) *E. diaphana*, and (C) *C. xamachana*, as well as a probability table detailing the likelihood of mitochondrial signaling peptide with predicted cleavage sites.

Predictions for *P. acuta* indicate a high mitochondrial targeting probability (0.99) with a predicted cleavage site between aa 28–29 (VGF-ST; Fig S3A). *E. diaphana* showed a similarly strong probability (0.97) with a predicted cleavage site also between its aa 28–29 (AXF-ST; Fig S3B), and *C. xamachana* yielded a 0.99 probability with a cleavage site between residues 26–27 (ASF-ST; Fig S3C). In all cases, the predicted N-terminal transit peptide and corresponding cleavage positions align with canonical mitochondrial targeting motifs observed in metazoan HSP60s. These consistent, high-confidence predictions (e.g., 0.99 probability for *P. acuta* and *C. xamachana*, 0.97 for *E. diaphana*) support that the cnidarian HSP60 sequences encode mitochondrial chaperonins and reinforce the evolutionary conservation of subcellular localization within this protein family.

## Discussion

Our findings demonstrate that mitochondrial HSP60 is deeply conserved across at least three cnidarian classes and maintains clear mechanistic continuity with its mammalian homolog. Using an anti-HSP60 monoclonal antibody (IgG mouse clone LK-2, Thermo Fisher Scientific) designed for mammalian systems, we successfully detected HSP60 in three ecologically and phylogenetically distinct cnidarians—*P. acuta*, *E. diaphana*, and *C. xamachana*—establishing functional cross-reactivity and supporting deep evolutionary conservation (Figs 2 and 3). Pairwise sequence alignments identified a 37-residue motif with high identity to human HSP60 (∼86–89%) and even greater conservation among cnidarians (∼92–95%) (Figs 4A–C and S1A and B). AlphaFold3-based structural mapping localized this motif to the equatorial domain in an equivalent position across taxa, consistent with antibody recognition and strong structural constraint (Fig 5). These data confirm that HSP60’s core mitochondrial chaperon function, in partnership with HSP10, is conserved from cnidarians to mammals, in agreement with previous observations in metazoan and bacterial studies (Martin et al, 1992; Höhfeld & Hartl, 1994; Dilly et al, 2012; Saibil, 2013; Voos, 2013; Bie et al, 2020). This conservation of the domain, which mediates ATP binding and oligomerization in Group I chaperonins, further provides strong evidence that the co-chaperonin HSP10 exists and functions equivalently in these cnidarians, consistent with deep mechanistic continuity across Metazoa. Conservation of this domain implies that secondary roles of HSP60, such as those involved in apoptosis, immune modulation, and unfolded-protein responses, likely originated early in animal evolution (Brocchieri & Karlin, 2000; Moré et al, 2001; Chandra et al, 2007; Cappello et al, 2019; Caruso Bavisotto et al, 2020).

Across experiment systems, HSP60 regulation under heat stress was context-dependent rather than uniform. To ensure experimental consistency across taxa, we applied a standardized increase in +5°C relative to the baseline husbandry temperature used for each organism. We acknowledge that this experimental increase was not intended to reproduce the exact thermal conditions of each species’ natural habitat. However, because all organisms were maintained under controlled laboratory conditions rather than sampled directly from the field, their baseline temperatures were based on established husbandry practices tailored to each taxonomic group represented in the study. To standardize the in vivo comparison across taxa, sampling was performed at three shared time points (T0, T12, and T24) following an acute +5°C increase above each species’ laboratory-maintained baseline temperature. Although this design enabled direct comparison across systems, the relatively coarse temporal resolution may have missed transient or rapidly changing HSP60 responses that occurred between sampling intervals. Within these constraints, *P. acuta* tissue lysates did not show reproducible detection of HSP60 expression, *E. diaphana* showed a progressive increase in HSP60 signal over time under heat stress, and *C. xamachana* maintained constitutive expression with temperature-dependent variation in signal intensity. In contrast, dissociated *P. acuta* cells exhibited variable HSP60 expression under both control and heat-stress conditions, further suggesting that HSP60 dynamics may depend on both temporal resolution and biological context. Table S1 summarizes HSP60 detection patterns across all experiments for better interpretation of these results. In addition, the statistical analyses indicate that HSP60 regulation was not uniform across the systems examined. The strongest statistical support was observed in *E. diaphana*, whereas *C. xamachana* showed a temperature-associated pattern. In *P. acuta*, HSP60 was detectable in dissociated cells but showed no significant changes across conditions and was not detected in tissue samples. These findings support a context-dependent interpretation of HSP60 expression across cnidarian systems.

β-actin band intensity varied across certain samples, especially in *P. acuta* tissue, making normalization to the loading control less reliable for analysis purposes. This variation may reflect the effects of heat stress on actin expression (Malerba et al, 2010; Yin et al, 2020). We, therefore, used total-protein normalization as an additional approach to interpret relative HSP60 band intensity. Although recombinant HSP60 was successfully detected in blots containing *P. acuta* tissue lysates, a formal recombinant calibration series was not performed to define the assay’s lower limit of detection or limit of quantification in this matrix. Moreover, whereas HSP60 was not reproducibly detected in the analyzed tissue samples, a HSP60 band was observed in one heat-stressed *P. acuta* tissue replicate during assay optimization under the same treatment conditions. This suggests that HSP60 may be present at low or variably detectable levels in coral tissue. Therefore, the absence of a consistent HSP60 signal in *P. acuta* tissue is interpreted here, conservatively, as a lack of detection under the present experimental conditions, rather than as definitive evidence of protein absence. Whereas further research is needed in in vitro cnidarian cellular biology, we propose that these differences in *P. acuta* HSP60 expression are best explained by biological rather than technical factors. In dissociated cell samples, tissue-level signaling, metabolic coupling, and intracellular feedback loops are disrupted, which we hypothesize leads to altered stress perception and loss of physiological buffering within the organism (Wallraff et al, 2006; O’Flanagan et al, 2019; Machado et al, 2021). Consequently, equivalent thermal challenges may trigger adaptive HSP60 induction in isolated cells compared with intact tissue. Comparable phenomena are observed in mammalian systems, where cell detachment affects HSF1 activation and alters HSP60 and HSP70 induction because of disrupted integrin signaling and mitochondrial instability (Gorrini et al, 2005; Kmiecik et al, 2020; Labbadia, 2023; Seluzicki et al, 2025). This cross-system parallel suggests that HSP60 regulation is fundamentally dependent on integrated physiological context, tissue organization, and taxa rather than temperature alone, supporting a hierarchical model of stress regulation that is altogether context-, lineage-, and temperature-dependent. For the thermally sensitive *P. acuta*, we hypothesize that the absence of detectable HSP60 induction in intact tissue fragments suggests that physiological buffering or successful tissue integrity signaling may suppress the immediate initiation of mitochondrial stress and secondary stress pathways (including those involving HSP60’s roles in apoptosis and immune modulation). This suppression, however, prevents the deployment of a critical molecular defense mechanism necessary to manage proteostatic demand, thereby contributing to the species’ thermal sensitivity and increased bleaching susceptibility. Future studies should incorporate recombinant HSP60 spike-in assays as well as pharmacological induction approaches to establish appropriate positive controls for HSP60 detection in cnidarian tissues.

The sea anemone *Exaiptasia* spp. (previously *Aiptasia*) exemplifies this hierarchical organization of stress regulation across environmental contexts and symbiotic states. Previous studies show that *E. diaphana* engages canonical signaling pathways such as TGF-β, NF-κB, and p53 during temperature- and salinity-driven stress (Vidal-Dupiol et al, 2014; Weizman & Levy, 2019; Eliachar et al, 2022; Labbadia, 2023). Under thermal extremes, this species exhibits cell-type- and symbiotic-stage–dependent transitions from apoptosis to necrosis, accompanied by HSP up-regulation that likely mitigates programmed cell death (Dunn et al, 2004). Environmental salinity further modulates these trajectories: whereas hypersalinity is generally deleterious in corals (often reducing photosynthetic efficiency and inducing bleaching) (Kerswell & Jones, 2003; Gardner et al, 2016; Randle et al, 2020) moderate-to-high salinity in *Exaiptasia* reduces symbiont loss and heat-induced bleaching, correlating with microbiome shifts that enhance nitrogen retention and antioxidant capacity (Randle et al, 2020). During prolonged heat exposures (e.g., 34°C for 28 d), stress pathways diverge by symbiotic state, indicating that oxidative stress is jointly governed by symbiotic state and organismal integration level (Weizman & Levy, 2019).

Jellyfish of the genus *Cassiopea* provide a complementary perspective on thermal adaptation. Living in shallow mangrove habitats characterized by strong diel temperature fluctuations, *C. xamachana* is physiologically pre-conditioned to thermal variability. Previous work reports minimal differential HSP expression under acute thermal stress and enhanced thermotolerance following acclimation to ∼32°C (Maloney et al, 2024). Similarly, *Cassiopea andromeda* maintains symbiosis during long-term exposure to ∼ +6°C, with high chlorophyll *a* content in symbionts indicating sustained photosynthetic function (Randle et al, 2020). Nevertheless, bleaching can occur under prolonged or extreme regimes: *C. xamachana* polyps’ bleach at 35°C for 2 wk, and adults at 34°C for 1 wk exhibit reduced bell diameters and partial bleaching, although reports of *Cassiopea* bleaching in the field remain scarce (McGill & Pomory, 2008; Djeghri et al, 2019; Banha et al, 2020; Newkirk et al, 2020). Comparisons with other scyphozoans, such as *Aurelia labiata* (ephyrae), which exhibit growth and swimming impairments at only +1.5°C above optimum, underscore the exceptional thermotolerance of *Cassiopea* spp. (Aljbour et al, 2019). The constitutive and temperature-dependent HSP60 signal observed in *C. xamachana* aligns with its life-history-driven thermal resilience.

Together, our results reveal a unifying pattern: HSP60’s amino acid sequence and domain architecture are evolutionarily conserved from cnidarians to mammals, but their regulatory dynamics are modulated by lineage, stress regime (intensity, duration, modality), and the level of biological organization. This context dependence is biologically meaningful because HSP60 abundance reflects mitochondrial integrity, free radical balance, and proteostatic demand (Singh et al, 2024). The transient HSP60 signal found in heated *P. acuta* cells likely reflects fluctuations in mitochondrial import efficiency or membrane potential with heat stress onset (Dimos et al, 2019), whereas the absence of signal in *P. acuta* fragments under heat stress confirms a context-dependent suppression mechanism that contributes directly to the species' thermal sensitivity and high susceptibility to bleaching (McRae et al, 2021). Thus, HSP60 functions not only as an evolutionarily ancient chaperone but also as a sensitive biomarker for mitochondrial stress and biological organization.

Importantly, our findings highlight the interpretive value of negative and variable results. Whereas strong HSP induction under severe stress is well documented in scleractinian corals, few studies explicitly report conditions under which HSP induction fails (Nakamura et al, 2011; Seveso et al, 2014, 2016, 2020). Documenting cases of non-induction, such as in *P. acuta* fragments and dissociated cells exposed to +5°C above optimum (variation in expression over exposure time), is essential for defining boundaries on HSP60’s reliability as a stress biomarker and improving reproducibility in coral stress research (Louis et al, 2025).

The future work should integrate protein- and transcript-level measurements (e.g., qRT-PCR, RNA-seq), employ targeted proteomics for absolute quantification, and localize HSP60 via immunofluorescence using mitochondrial markers and peptide-blocking controls. Physiological assays for free radical generation, mitochondrial membrane potential, ATP/ADP ratio, and intracellular pH, combined with apoptosis markers (caspase activity, TUNEL), would clarify the mechanistic basis of HSP60 regulation. Implementing ecologically realistic stress paradigms, such as gradual temperature ramps, fluctuating salinity and pH, and altered photoperiod, under defined symbiotic states, coupled with comparative genomics (orthology validation, genome-wide dN/dS, and site-specific constraint analyses), will help delineate when HSP60 reliably indicates mitochondrial stress versus regulatory fine-tuning. Such integrative approaches will refine the application of HSP60 as a mechanistic biomarker for predicting symbiosis stability and bleaching susceptibility under ocean warming.

## Materials and Methods

### Study organisms overview

The organisms used in this study are the scleractinian coral *P. acuta* (green phenotype), sea anemone *E. diaphana* (H2 clone line), and the upside-down jellyfish *C. xamachana* (Fig 1A–C). *P. acuta* is considered to be a thermally sensitive species, with documented bleaching responses at temperatures 1–2°C above local summer maxima (Roper et al, 2023; García et al, 2024). The symbiont population in *P. acuta* is typically composed of *Cladocopium goreaui* (C1), with a low relative abundance of *Durusdinium* type D1 (Ros et al, 2021; Lin et al, 2024; Millán-Márquez et al, 2024; Duijser et al, 2025). The colonies used in this study belong to a monoclonal population originating from Hawaii and propagated in aquaculture for research purposes (Putnam Lab, University of Rhode Island, USA).

The glass anemone *E. diaphana* is a small, pale brown, symbiotic anemone, which has been widely used as a model organism for understanding cnidarian symbiosis and physiology (Jiang et al, 2021). This species is commonly associated with the dinoflagellate *Breviolum minutum* (formerly Clade B) but is capable of hosting other *Symbiodiniaceae* genera under experimental conditions, e.g., *Symbiodinium* spp., *Durusdinium* spp. (LaJeunesse et al, 2018; Tortorelli et al, 2020). H2 clone line individuals were originally shared with us from the Jinkerson Lab (University of California, Riverside, USA).

The upside-down jellyfish, *C. xamachana*, is a rising model system for investigating cnidarian-algal symbiosis, tissue regeneration, and evolutionary biology. Recent investigations highlight the use of *Cassiopea* spp. organisms as indicator species for environmental monitoring and ecotoxicology and virology (Klein et al, 2016; Mirshamsi et al, 2017; Ohdera et al, 2018; McKenzie et al, 2020; Olguín-Jacobson & Pitt, 2021). *C. xamachana* is mostly found in the Western Atlantic Ocean, Caribbean Sea, Gulf of Mexico, and Florida Keys (specimens used here) and hosts *Symbiodinium microadriaticum* (clade A1). *S. microadriaticum* infection is essential to *Cassiopea* spp. life cycles (Morejón-Arrojo et al, 2025). Polyps of *C. xamachana* were originally shared with us from the Buckley Lab (Auburn University, USA).

### Animal husbandry

Colonies of *P. acuta* (green phenotype) were kept in 170 L aquaria (with 24 L sump), supplied with artificial seawater (ASW, 35 ppt salinity; Tropic Marin Pro-Reef Salt), maintained at 25°C (600 W titanium aquarium heater with Inkbird ITC–306 A InkBird temperature controller), under a 12 h: 12 h photoperiod at 150–200 μmol photons m^−2^ s^−1^ irradiance (AP9x LED lights; Kessil). The ASW was constantly filtered (200 μm sock filters; Aquatic Experts), skimmed for organics (Octo Classic 110S protein skimmer; Reef Octopus), and UV sterilized (Aqua Ultraviolet). Seawater parameters (pH, redox potential, temperature, carbonate hardness [kH], calcium, magnesium, phosphate, and nitrate levels) were monitored daily (Proflux 4 GHL and API saltwater test kits) and adjusted using dosing buffers when needed (Fauna Marin Balling Light Set; Fauna Marin). Corals were fed once a week with live *Artemia salina* nauplii (E-Z EGG; Brine Shrimp Direct). Aquaria were cleaned once a week to limit biofouling. A 10% water change was also performed weekly. The colonies used in this study belong to a monoclonal population originating from Hawaii and propagated in aquaculture for research purposes (Putnam Lab, University of Rhode Island).

Specimens of *E. diaphana* (H2 clone line) were maintained in Bisphenol-A-free (BPA-free) clear polypropylene containers (Cambro) supplied with ASW (salinity of 35 ppt; Tropic Marine Pro-Reef salt), maintained at 22°C, and irradiance of 200 μmol photons m^−2^ s^−1^ on a 12 h:12 h photoperiod (Fluval Marine Nano LED light). The anemones were fed with live *A. salina* napulii once a week. The containers were cleaned, and the ASW was renewed with a weekly 100% water change to remove debris, prevent algal growth, and prevent microbial contamination, thereby maintaining optimal conditions for growth and survival of the organisms.

Stock cultures of medusae of *C. xamachana* (non-clonal) were maintained in 170 L aquaria (with 24 L sump) supplied with ASW (38 ppt salinity; Tropic Marin Pro-Reef Salt), maintained at 27°C (600 W titanium aquarium heater with Inkbird ITC–306 A InkBird temperature controller), under a 12 h: 12 h photoperiod at 150–200 μmol photons m^−2^ s^−1^ irradiance (AP9x LED lights; Kessil). The ASW was constantly filtered (200 μm sock filter; Aquarium Experts), skimmed for organics (OCTO classic 110 SSS protein skimmer; Reef Octopus), and UV sterilized (Aqua Ultraviolet). The seawater parameters (pH, redox potential, temperature, carbonate hardness, calcium, magnesium, phosphate, and nitrate levels) were monitored daily (Profilux 4; GHL and API saltwater test kits) and adjusted using buffers when needed (Fauna Marin Balling Light Set; Fauna Marin). The polyps of *C. xamachana* were grown in a separate container (BPA-free clear Cambro), under the same conditions described above. The polyps were fed with live *A. salina* nauplii (E-Z EGG; Brine Shrimp Direct) twice a week, and the medusae were fed daily.

### Experimental design

Two complementary approaches were used to assess HSP60 regulation: in vitro assays using dissociated cells from *P. acuta* to capture cellular-level responses, and in vivo, whole-organism experiments from which tissue slurries were prepared using *P. acuta*, *C. xamachana*, and *E. diaphana*.

In vitro assays were performed by exposing dissociated cells to control and heated conditions (+5°C above ambient aquaria temperature) in lit cell culture incubators (12–12 h photoperiod). In vivo experiments were conducted by transferring the whole organisms from their ambient aquaria directly to a heated experimental tank, with a temperature of +5°C above ambient aquaria temperature: *P. acuta* control = 25 ± 0.5°C versus heated = 30 ± 0.5°C, *E. diaphana* control = 22 ± 0.5°C versus heated 27 ± 0.5°C, and *C. xamachana* control 27 ± 0.5°C versus heated = 32 ± 0.5°C. Dissociated cells, tissue slurries, and lysates were obtained as described next.

### Coral cell dissociation and cell sample collection

The coral cell dissociation protocol (Roger & Chowdhury, 2026) is adapted from Roger et al (2021). A single nubbin from the *P. acuta* colony (∼1 cm length) was cut using sterile clippers and transferred to a crystallization dish containing ASW with ReefDip coral disinfectant (25 μl per ml of ASW) under constant bubbling for 10 min. The nubbin was then rinsed with sterile-filtered artificial seawater (FASW) (0.22 μm) thrice and incubated in sterile-filtered calcium-magnesium-free artificial seawater (CMFASW, 0.22 μm) for 1 h in a biosafety cabinet under ambient light. Post incubation, the nubbin was gently washed with CMFASW to detach cells from the surface of the nubbin. The cell suspension was centrifuged at 204*g* for 3 min at 25°C. The supernatant was removed and the cells resuspended in complete coral cell culture media (15% DMEM without phenol red, Catalog no. 21063029, 10% FBS; Thermo Fisher Scientific, Catalog no. A5670701, 0.5%, antibiotics-antimycotics; Thermo Fisher Scientific, Catalog no. 15240062, 0.5% Gentamicin; Thermo Fisher Scientific, Catalog no. 15710064, and 74% sterile FASW; Thermo Fisher Scientific) The coral cell suspension was collected in microcentrifuge tubes every 6 h for a total duration of 24 h. The cells were centrifuged at 6,010*g* at 4°C for 10 mins. The anemone and jellyfish sample specimens were collected every 12 h, homogenized, and centrifuged at 6,010*g* at 4°C for 10 min to collect the tissue pellets, which were maintained at −20°C until further use. Throughout the study, n = 3 denotes biological replication. In *P. acuta* fragment experiments, each replicate was derived from a different colony. In the corresponding cell-based experiments, each replicate consisted of an independent cell preparation from a separate coral fragment (nubbin) and was normalized to 1 × 10^6^ cells ml^−1^ at each sampling point. For *E. diaphana*, replicates were based on pooled samples from six independent individuals, whereas for *C. xamachana*, each replicate represented a single, independently collected medusa.

### Tissue homogenization

Once the thermal experiment was completed, individuals of *E. diaphana* (∼5 mm) and *C. xamachana* (∼2 cm in diameter) were transferred to a 50 ml conical tube containing 4 ml FASW and homogenized (handheld motor) for 10–15 s intervals on ice to prevent protein degradation. The homogenate was centrifuged at 6,010*g* for 10 min at 4°C. The supernatant was removed, and the cell pellets were stored in −20°C until protein extraction.

### Total-protein extraction and analysis

The total-protein extraction for dissociated cells from the *P. acuta* fragment was adapted from Seveso et al (2014), with minor modifications. The cells were resuspended in 100 μl of lysis buffer (0.0625 M Tris–HCl, pH 6.8, 10% glycerol, 0.1% wt/vol SDS, 1% wt/vol 2-mercaptoethanol containing 1 mM phenylmethylsulfonyl fluoride, and complete EDTA-free cocktail of protease inhibitors). The cells with the lysis buffer mixture were boiled for 10 min at 95°C and centrifuged at 13,500*g* for 15 min at 4°C. The supernatant was further clarified by centrifuging again (2,350*g* for 5 min).

The total-protein concentration was determined using the Bradford Assay, and the absorbance was measured using a microplate reader (595 nm).

The protein samples were separated by sodium dodecyl sulfate-polyacrylamide gel electrophoresis (SDS–PAGE), using 10% polyacrylamide gels; 20 μg of total protein was loaded into each lane of the gel. Two gels were run simultaneously; one gel was stained with InstantBlue Coomassie Protein Stain to visualize protein bands, and the other gel was used for the western immunoblot assay. Recombinant HSP60 was included as a positive control in the relevant western blot assays, including those performed with *P. acuta* tissue lysates. Total-protein concentration was measured before loading using the Bradford assay (Table S5). The PVDF membrane (0.2 μm) was stained with Ponceau S staining solution to confirm protein transfer on the membrane. Ponceau S is a staining solution that provides a nondestructive, reversible way to stain membranes and confirm protein transfer without disrupting the integrity of the target proteins. The membrane was incubated in 5% Blocking solution (5% nonfat dry milk in 1X TBST: Tris-buffered saline, 0.1% Tween-20) for 1 h. After blocking, the membrane was incubated with anti-HSP60 monoclonal antibody (IgG mouse clone LK-2) at a 1:1,000 dilution, and the membrane was triple-washed with 1X TBST buffer and incubated with horseradish peroxidase conjugated-anti-mouse IgG secondary antibody, 1:10,000 dilution in 5% blocking solution for 2 h. The chemiluminescence signal was detected using Pierce ECL Western blotting substrate and imaged using a gel imager. The membranes were stripped, reblocked, and incubated with anti-actin monoclonal antibody (clone C4) at a 1:3,000 dilution overnight at 4°C. The membrane was triple-washed with 1X TBST buffer and incubated with horseradish peroxidase conjugated-anti-mouse IgG secondary antibody, 1:10,000 dilution in 5% blocking solution for 2 h. The chemiluminescence signal was detected using Pierce ECL western blotting substrate and imaged using a gel imager.

Table S5. Protein concentrations of samples used for western blotting across organisms, time points, and temperature treatments, reported in mg/ml and μg/ml.

Band intensities were quantified using ImageJ/Fiji. Original chemiluminescent blot images were opened without contrast enhancement for measurement. A uniform rectangular region of interest was drawn around each band, and the same region of interest size was maintained for all lanes on a given blot. Integrated density was measured for each target and loading-control band. To correct for the nonspecific signal, the background was measured from an adjacent membrane region lacking visible bands and subtracted from the raw band intensity. The corrected HSP60 signal for each lane was normalized to the corrected β-actin signal from the same lane. The resulting normalized values were used as relative densitometric measurements. Because a formal dilution-series experiment was not performed, the linear dynamic range of detection for HSP60 and β-actin was not explicitly validated here.

### Statistical analysis

Each experimental system was analyzed separately to reflect differences in study design among species and sample types, rather than combining all datasets into a single cross-species model. For datasets with measurable signal and replication, normalized HSP60 values were analyzed using two-way ANOVA with temperature, time, and the temperature: time interaction as fixed effects. For *E. diaphana* and *C. xamachana*, only time points common to both temperature treatments were included. For each analysis, n refers to independent biological replicates (n = 3 per condition), where each replicate corresponds to a separate organism or independent cell preparation maintained in an individual well. Technical replicates were averaged before analysis. Two-way ANOVA models included temperature (two levels) and time (three or five levels, depending on the dataset), and the degrees of freedom reflect the number of factor levels and total independent observations included in each model. Model assumptions were evaluated by examining residuals and by applying Shapiro–Wilk tests for normality and Levene’s tests for homogeneity of variance. Exact *P*-values are reported where relevant, and statistical significance was set at *P* < 0.05. For *P. acuta* tissue samples, normalized HSP60 values were uniformly zero across all conditions. Because there was no measurable variation in the response variable, formal ANOVA was not informative for this dataset, and the results were interpreted descriptively. Each experimental system was analyzed independently with predefined comparisons; therefore, no formal correction for multiple testing was applied; results are interpreted within each system rather than across all analyses. Given the small sample size (n = 3), effect sizes were not emphasized and results are reported primarily using F-statistics and *P*-values.

### Bioinformatics

#### Multiple sequence alignment

To assess evolutionary conservation and sequence homology of HSP60 across representative cnidarian taxa, MSA using pairwise alignment was performed on NCBI BLASTp using HSP60 amino acid sequences derived from *P. acuta*, *E. diaphana*, and *C. xamachana*, alongside the canonical human HSP60 (HSPD1, UniProt ID: P10809) as reference. The candidate coral (*P. acuta*) sequences were obtained from Vidal-Dupiol et al (2020) *Preprint*, *C. xamachana* sequences from the Joint Genome Institute, and *E. diaphana* sequences from ReefGenomics (Kayal et al, 2018; Newkirk et al, 2022). The results from this alignment provided orthologs for *P. acuta* (596 aa), *E. diaphana* (586 aa), and *C. xamachana* (583 aa). The pairwise alignments show a highly conserved amino acid sequence within the C-terminal region (alignment ranges 391–434). The identities for these candidate HSP60 sequences are 85% (*P. acuta* and *E. diaphana*) and 89% (*C. xamachana*), with 100% positives and no gaps present (E-values between 8 × 10^−20^ and 6 × 10^−20^).

Protein identity and orthology were confirmed via BLASTp (NCBI) against the non-redundant database, retaining top hits with query coverage >80% and E-value <1 × 10^−5^. Conserved residues and functional domains were annotated based on the human HSP60 reference sequence mentioned above.

#### Epitope alignment

For antibody epitope mapping, the experimentally validated linear epitope of human HSP60 (mouse, monoclonal LK-2, MA5-45114; Thermo Fisher Scientific) was obtained from product datasheets (Thermo Fisher Scientific). The corresponding epitope region (aa 383–419) was aligned to the cnidarian HSP60 orthologs within the MSA framework to identify conserved and variable residues potentially contributing to antibody recognition. Phylogenetic relationships among cnidarian HSP60 sequences and the human HSPD1 reference were to be inferred using MEGA X (Sievers & Higgins, 2014) under the maximum-likelihood (ML) method with default parameters. Branch support was assessed using 1,000 bootstrap replicates.

#### AlphaFold3 structural prediction and epitope mapping

Predicted tertiary structures of HSP60 homologs from *P. acuta* (accession ID: TCONS_00030188), *C. xamachana* (accession ID: Casxa1|9735), and *E. diaphana* (accession ID: AIPGENE15267 [P18687]) were obtained from the AlphaFold Protein Structure database (DeepMind and EMBL). For each species, the highest-ranked model was downloaded (AlphaFold Version 3) in CIF format and visualized in UCSF ChimeraX (Version 1.10.1). Per-residue confidence scores were derived from the predicted local distance difference test (pLDDT) values.

Conserved regions corresponding to the human HSP60 epitope (aa 383–419) were identified using Clustal Omega multiple sequence alignment on NCBI BLASTp. Pairwise alignments using the query 37 amino acid epitope from *P. acuta* show strong conservation in *E. diaphana* (35/37 identities; 95% identity; 100% positives; 0 gaps; E-value 8 × 10^−24^) and *C. xamachana* (34/37 identities; 92% identity; 100% positives; 0 gaps; E-value 2 × 10^−2^). Homologous residues were mapped to each cnidarian HSP60 sequence (positions 398–434 in *P. acuta*, 393–429 in *E. diaphana*, and 391–427 in *C. xamachana*) and highlighted on their respective AlphaFold-predicted tertiary structures. These residues were rendered in cartoon representations (cyan) to evaluate spatial conservation, solvent exposure, and structural context of the conserved epitope within the chaperonin architecture.

#### Mitochondrial protein prediction

To determine the subcellular localization of cnidarian HSP60 homologs, candidate protein sequences were analyzed using TargetP-2.0 (DTU Health Tech). Sequences from *P. acuta* (TCONS_003018), *E. diaphana* (AIPGENE15267 [P18687]), and *C. xamachana* (Casxa1|9735) were submitted as the queries to predict mitochondrial targeting peptides. TargetP-2.0 provides the probability of mitochondrial localization and identifies N-terminal cleavage sites. Predicted probability scores and cleavage positions were recorded for each sequence and visualized as probability plots of amino acid position versus localization likelihood (Fig S3).

## Supplementary Material

Reviewer comments

## Data Availability

The protein sequences for the study organisms were obtained from Reef Genomics (http://reefgenomics.org/), Joint Genome Institute (https://jgi.doe.gov/), and NCBI (https://www.ncbi.nlm.nih.gov/). The detailed protocol for coral cell dissociation is available on Protocols.io (Roger & Chowdhury, 2026). The immunoblot quantification data, R scripts, and the summary of experiments are available on Open Science Framework (Chowdhury et al, 2026). All analyses were performed using R statistical software (Version 2025.09.1+401). Western blot quantification was performed in FIJI/ImageJ.
