# Supplementary figures and images for: Conserved HSP60 structure with lineage- and context-specific regulation in cnidarians
Source: Life Sci Alliance. 2026 Jun 24;9(9):e202503592. doi: 10.26508/lsa.202503592 (PMC13293977; doi:10.26508/lsa.202503592)

*P.acuta* fragment- Blot for HSP60

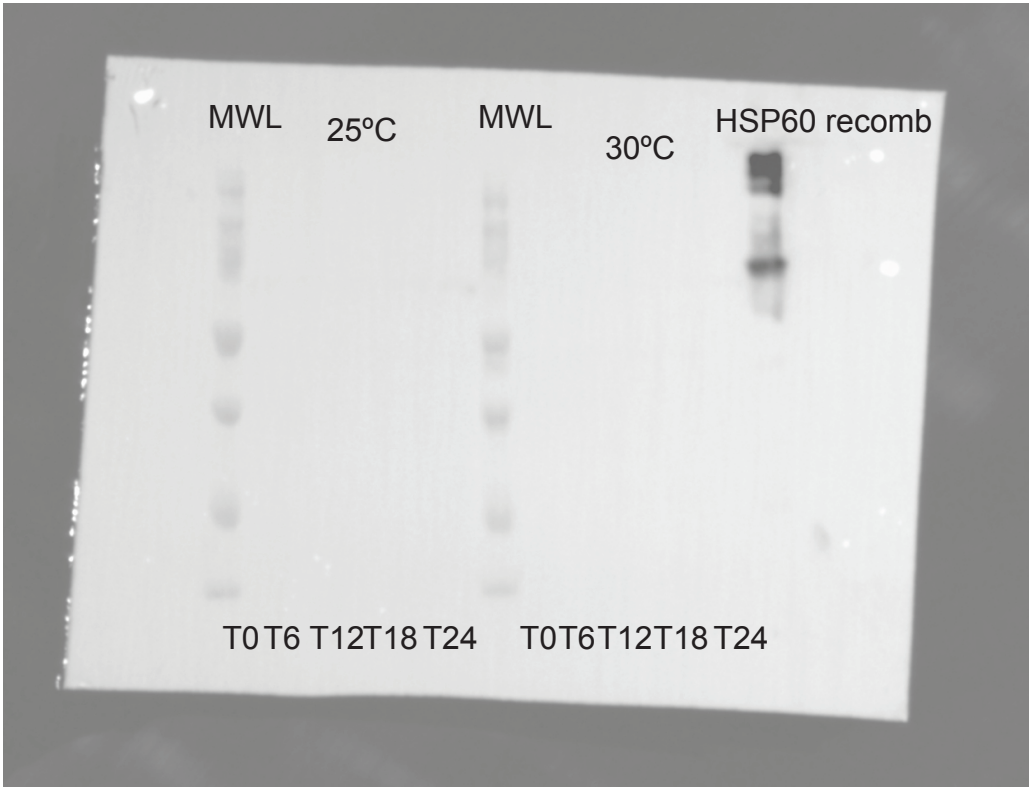

*P.acuta* fragment- Blot for Actin

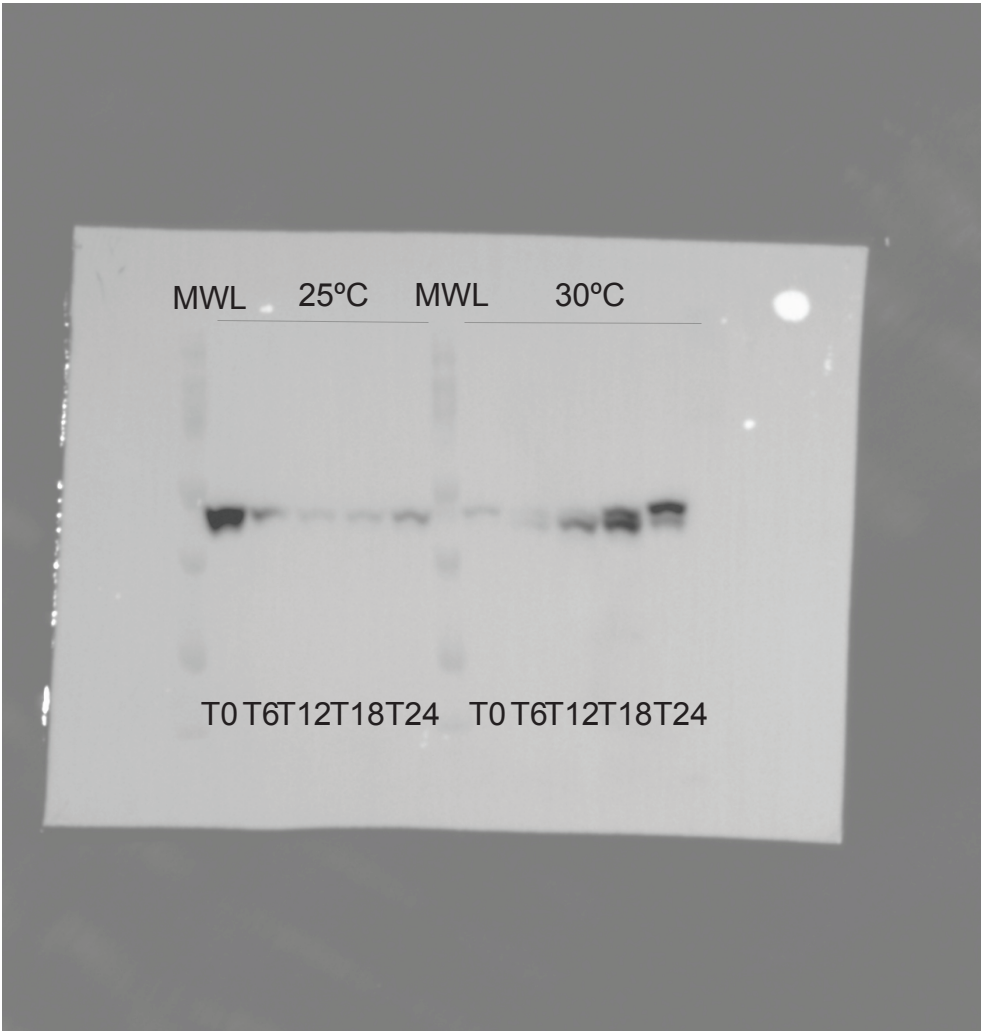

Supplement: Supplementary file 1 [file LSA-2025-03592_SdataF2.1.pdf]

*E.diaphana* HSP60 blot

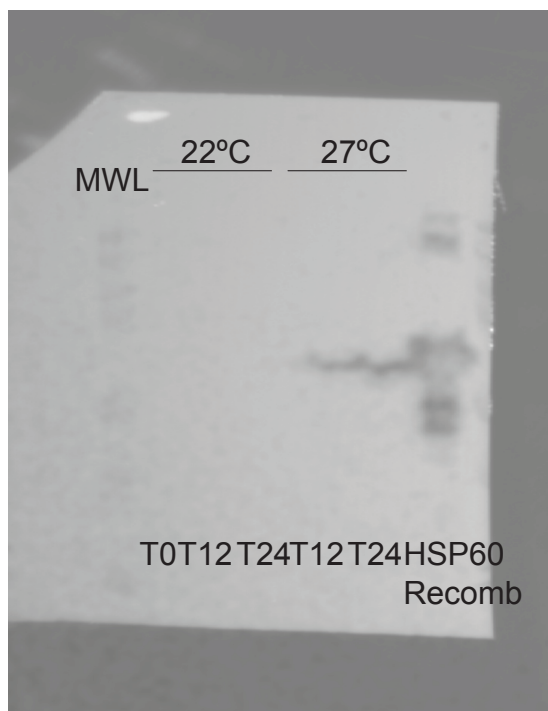

*E.diaphana* Beta-actin blot

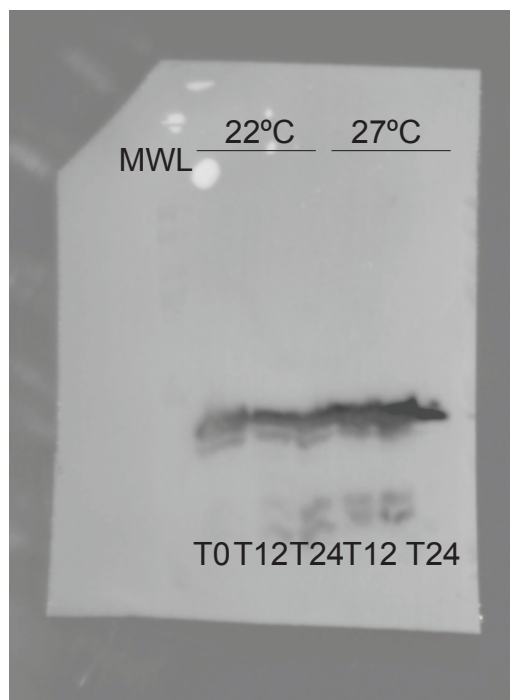

Supplement: Supplementary file 2 [file LSA-2025-03592_SdataF2.2.pdf]

*C.xamachana* HSP60 blot

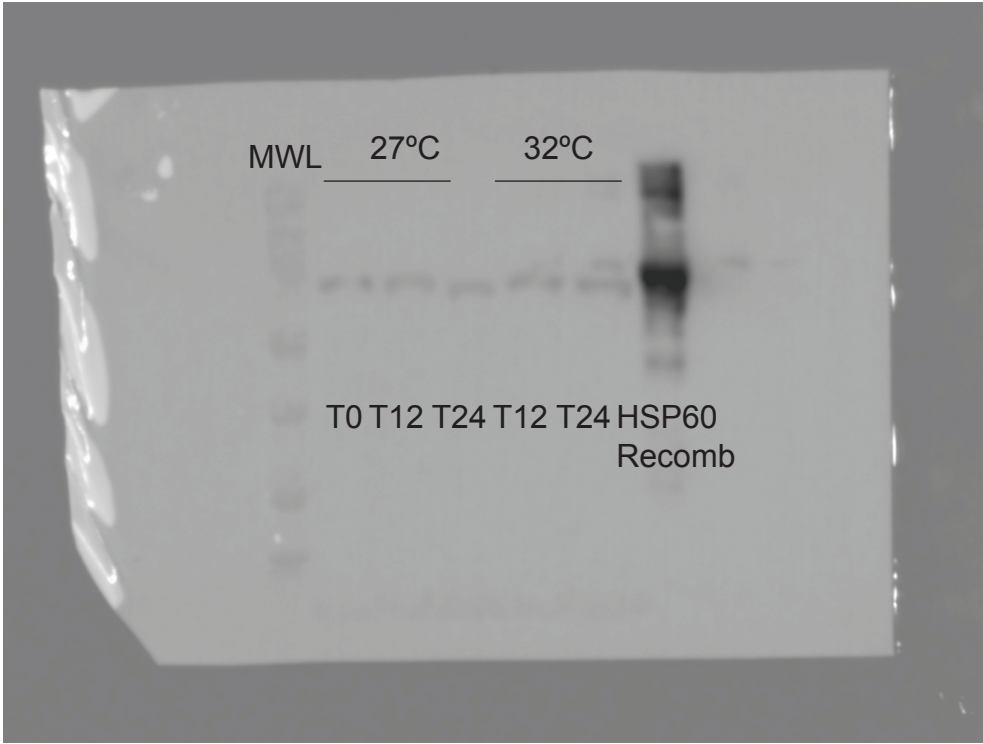

*C.xamachana* Beta-actin blot

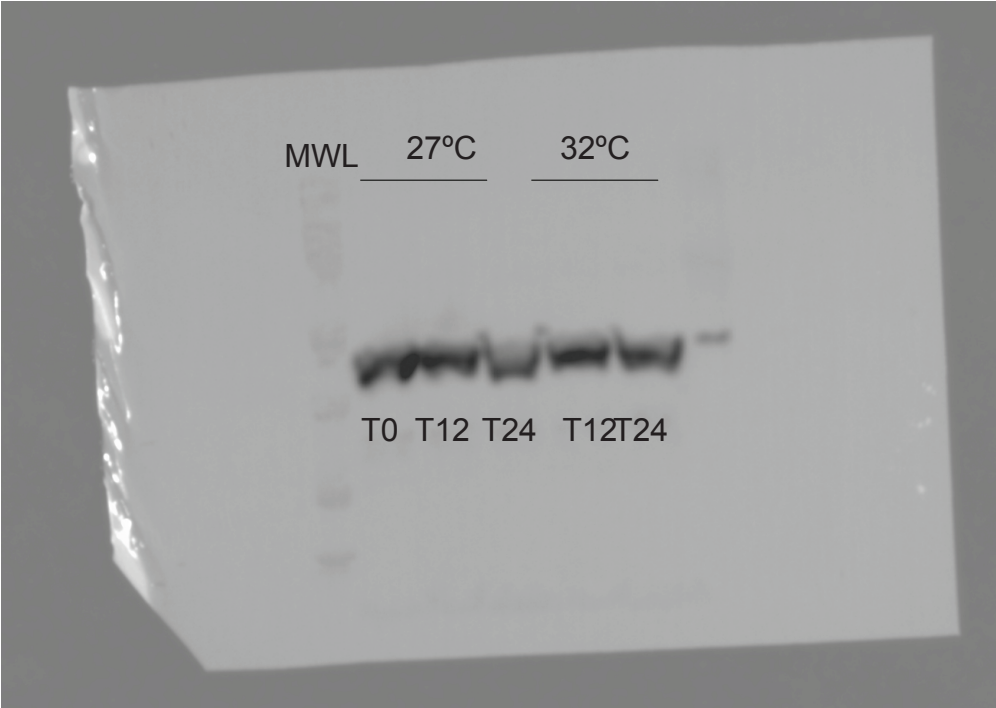

Supplement: Supplementary file 3 [file LSA-2025-03592_SdataF2.3.pdf]

*P.acuta* cell- Blot for HSP60 and Beta-actin

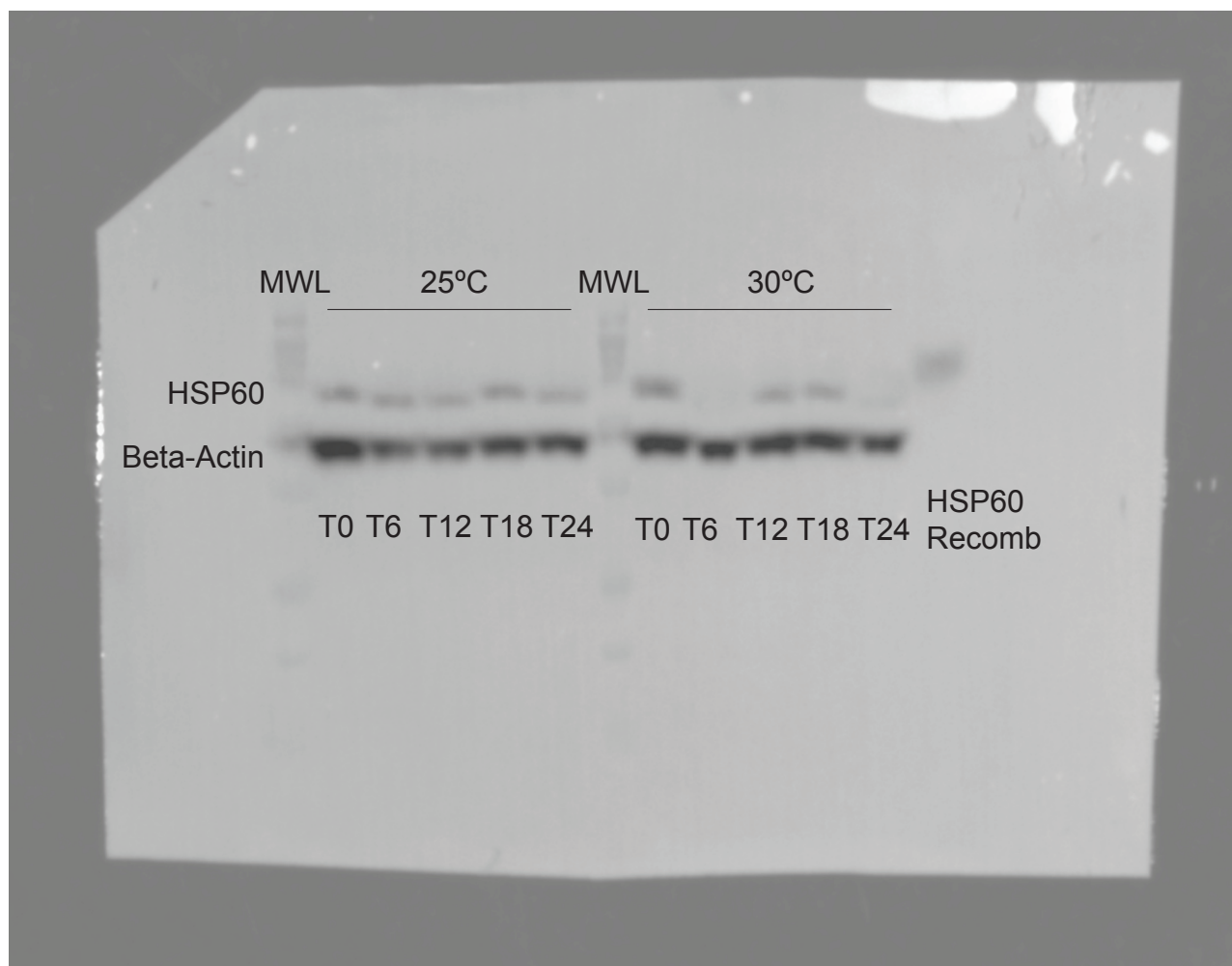

Supplement: Supplementary file 5 [file LSA-2025-03592_SdataF3.pdf]
