# Supplementary material for: Conserved HSP60 structure with lineage- and context-specific regulation in cnidarians
Source: Life Sci Alliance. 2026 Jun 24;9(9):e202503592. doi: 10.26508/lsa.202503592 (PMC13293977; doi:10.26508/lsa.202503592)
Supplement: Supplementary file 4 [file LSA-2025-03592_TableS1.docx]

**Table S1. Summary of HSP60 and β-actin detection across species, sample types, temperature treatments, and sampling time points.** Overview of HSP60 outcome and β-actin detection in *Pocillopora acuta*, *Exaiptasia diaphana*, and *Cassiopea xamachana* under different experimental temperature regimes and sampling intervals. Results are presented by species and sample type. “+” indicates positive detection, “−” indicates no detection, and “Mixed (+/−)” indicates inconsistent detection across samples or time points. β-actin served as the loading control.

| **Species** | **Sample type** | **Temperature** | **Time points** | **HSP60 outcome** | 𝛃**-actin**  **outcome** |
| --- | --- | --- | --- | --- | --- |
| *P. acuta* | Cell | 25°C | 0h only | + | + |
| *P. acuta* | Cell | 25°C and 30°C | 0, 6, 12, 18, 24h | Mixed  (+/-) | Mixed (+/-) |
| *P. acuta* | Cell | 32°C | 0, 6, 12, 18, 24, 30, 36, 42, 48h | + | + |
| *P. acuta* | Cell | 25°C and 33°C | 0, 6, 24, 48, 72h | - | - |
| *P. acuta* | Cell | 25°C and 30°C | 0 and 24h | - | + |
| *P. acuta* | Fragment | 25°C and 30°C | 0, 12, 24h | Mixed  (+/-) | Mixed (+/-) |
| *P. acuta* | Fragment | 25°C and 30°C | 0, 6, 12, 18, 24h | - | + |
| *E. diaphana* | Whole organism/tissue | 27°C | 0, 12, 24h | + | + |
| *E. diaphana* | Whole organism/tissue | 33°C | 0, 12, 24, 36, 48h | + | + |
| *E. diaphana* | Whole organism/tissue | 33°C | 0 and 7 days | Mixed  (+/-) | + |
| *E. diaphana* | Whole organism/tissue | 30°C and 35°C | 0, 7, and 8 days | + | + |
| *C. xamachana* | Whole organism/tissue | 32°C | 0, 12, 24h | + | + |
| *C. xamachana* | Whole organism/tissue | 32°C | 0, 24, 48, 72h | Mixed  (+/-) | + |
| *C. xamachana* | Whole organism/tissue | 35°C | 0, 24, 48, 72h | Mixed  (+/-) | + |
