# Supplementary material for: Conserved HSP60 structure with lineage- and context-specific regulation in cnidarians
Source: Life Sci Alliance. 2026 Jun 24;9(9):e202503592. doi: 10.26508/lsa.202503592 (PMC13293977; doi:10.26508/lsa.202503592)
Supplement: Supplementary file 6 [file LSA-2025-03592_TableS2.docx]

**Table S2. Statistical comparison of normalized HSP60 expression across cnidarian systems under control and heat-stress conditions.** Table summarizing the results obtained from two-way ANOVA tests of HSP60 band intensity normalized to β-Actin. The significance codes are designated as: 0 ‘***’ 0.001 ‘**’ 0.01 ‘*’ 0.05 ‘.’ 0.1 ‘ ’ 1

| ***E. diaphana*: HSP60/ β-Actin** | | | | | |
| --- | --- | --- | --- | --- | --- |
| **Parameter** | **Df** | **Sum Sq** | **Mean Sq** | **F value** | **Pr (>F)** |
| Temperature | 1 | 0.5935 | 0.5935 | 5.405 | 0.0486 ***** |
| Time | 1 | 0.6282 | 0.6282 | 5.721 | 0.0437 ***** |
| Temperature: Time | 1 | 0.0926 | 0.0926 | 0.844 | 0.3852 |
| Residuals | 8 | 0.8784 | 0.1098 |  |  |
| ***E. diaphana*: HSP60/ Total protein** | | | | | |
| **Parameter** | **Df** | **Sum Sq** | **Mean Sq** | **F value** | **Pr (>F)** |
| Temperature | 1 | 0.367 | 0.367 | 4.311 | 0.0715 **.** |
| Time | 1 | 0.1049 | 0.1049 | 1.232 | 0.2992 |
| Temperature: Time | 1 | 0.0516 | 0.0516 | 0.606 | 0.4588 |
| Residuals | 8 | 0.6809 | 0.0851 |  |  |
| ***C.xamachana*: HSP60/ β-Actin** | | | | | |
| **Parameter** | **Df** | **Sum Sq** | **Mean Sq** | **F value** | **Pr (>F)** |
| Temperature | 1 | 1.3392 | 1.3392 | 16.129 | 0.00386 ** |
| Time | 1 | 0.0002 | 0.0002 | 0.003 | 0.96131 |
| Temperature: Time | 1 | 0.1209 | 0.1209 | 1.456 | 0.262 |
| Residuals | 8 | 0.6642 | 0.083 |  |  |
| ***C.xamachana*: HSP60/ Total protein** | | | | | |
| **Parameter** | **Df** | **Sum Sq** | **Mean Sq** | **F value** | **Pr (>F)** |
| Temperature | 1 | 0.1612 | 0.1612 | 4.685 | 0.0623 . |
| Time | 1 | 0.03388 | 0.03388 | 0.985 | 0.3501 |
| Temperature: Time | 1 | 0.00052 | 0.00052 | 0.015 | 0.9055 |
| Residuals | 8 | 0.27524 | 0.03441 |  |  |
| ***P.acuta*: HSP60/ β-Actin** | | | | | |
| **Parameter** | **Df** | **Sum Sq** | **Mean Sq** | **F value** | **Pr (>F)** |
| Temperature | 1 | 0.006 | 0.0064 | 0.019 | 0.893 |
| Time | 4 | 0.314 | 0.0784 | 0.228 | 0.92 |
| Temperature: Time | 4 | 0.59 | 0.1474 | 0.428 | 0.787 |
| Residuals | 20 | 6.891 | 0.3445 |  |  |
| ***P.acuta*: HSP60/ Total protein** | | | | | |
| **Parameter** | **Df** | **Sum Sq** | **Mean Sq** | **F value** | **Pr (>F)** |
| Temperature | 1 | 0.0518 | 0.05177 | 1.103 | 0.306 |
| Time | 4 | 0.0813 | 0.02034 | 0.433 | 0.783 |
| Temperature: Time | 4 | 0.0974 | 0.02435 | 0.519 | 0.723 |
| Residuals | 20 | 0.9387 | 0.04693 |  |  |
