# Supplementary material for: Conserved HSP60 structure with lineage- and context-specific regulation in cnidarians
Source: Life Sci Alliance. 2026 Jun 24;9(9):e202503592. doi: 10.26508/lsa.202503592 (PMC13293977; doi:10.26508/lsa.202503592)
Supplement: Supplementary file 7 [file LSA-2025-03592_TableS3.docx]

**Table S3. Table showing alignment of candidate sequences.**

Table comparing the sequence alignment of candidate HSP60 protein sequences within *P. acuta* (stony coral), *E. diaphana* (sea anemone), and *C. xamachana* (upside-down jellyfish) against the known human HSP60 (chaperonin-60) sequence (P10809).

| **Organism** | **Candidate sequence**  **(source)** | **Length** | **Score** | **E-value** | **Identities** | **Positives** | **Gaps** |
| --- | --- | --- | --- | --- | --- | --- | --- |
| *P. acuta* | TCONS_0030188  (Vidal-Dupiol et al, 2020) | 596 | 803 bits (2073) | 0 | 401/564 (71%) | 474/564 (84%) | 10/564 (1%) |
| *E. diaphana* | P18687  (Reef Genomics) | 586 | 808.52 (2087) | 0 | 394/566 (69.6%) | 480/566 (84.8%) | 10/566 (1.8%) |
| *C. xamachana* | Casxa1\|9735  (JGI) | 583 | 967 bits (2499) | 0 | 513/561 (91%) | 541/561 (96%) | 0/561 (0%) |
