# Supplementary material for: Conserved HSP60 structure with lineage- and context-specific regulation in cnidarians
Source: Life Sci Alliance. 2026 Jun 24;9(9):e202503592. doi: 10.26508/lsa.202503592 (PMC13293977; doi:10.26508/lsa.202503592)
Supplement: Supplementary file 8 [file LSA-2025-03592_TableS4.docx]

**Table S4. Table showing epitope alignments among candidate sequences.**

Table comparing the results generated from the epitope sequence of human HSP60 (p10809) epitope (amino acids 383-419) aligned with candidate HSP60 sequences in *P. acuta* (stony coral), *E. diaphana* (sea anemone), and *C. xamachana* (upside-down jellyfish).

| **Organism** | **Candidate sequence**  **(source)** | **Length** | **Score** | **E-value** | **Identities** | **Positives** | **Gaps** |
| --- | --- | --- | --- | --- | --- | --- | --- |
| *P. acuta* | TCONS_0030188  (Vidal-Dupiol et al, 2020) | 37 | 68.2 bits (165) | 3x10^-20^ | 32/37 (86%) | 37/37 (100%) | 0/37 (0%) |
| *E. diaphana* | P18687  (Reef Genomics) | 37 | 68.6 bits (166) | 2x10^-20^ | 32/37 (86%) | 37/37 (100%) | 0/37 (0%) |
| *C. xamachana* | Casxa\|9735  (JGI) | 37 | 68.6 bits (166) | 2x10^-20^ | 33/37 (89%) | 37/37 (100%) | 0/37 (0%) |
