# Supplementary material for: Conserved HSP60 structure with lineage- and context-specific regulation in cnidarians
Source: Life Sci Alliance. 2026 Jun 24;9(9):e202503592. doi: 10.26508/lsa.202503592 (PMC13293977; doi:10.26508/lsa.202503592)
Supplement: Supplementary file 9 [file LSA-2025-03592_TableS5.docx]

**Table S5. Protein concentrations of samples used for western blotting across organisms, time points, and temperature treatments, reported in mg/mL and µg/mL**

| **Organism** | **Samples** | **Protein concentration (mg/ mL)** | **Protein concentration µg/mL** |
| --- | --- | --- | --- |
| ***E. diaphana*** |  |  |  |
|  | T0-22ºC | 0.2 | 200 |
|  | T12-22ºC | 0.255 | 255 |
|  | T24-22ºC | 0.244 | 244 |
|  | T12-27ºC | 0.26 | 260 |
|  | T24-27ºC | 0.205 | 205 |
| ***C. xamachana*** |  |  |  |
|  | T0-27ºC | 1.73 | 1730 |
|  | T12-27ºC | 1.92 | 1920 |
|  | T24-27ºC | 2.531 | 2531 |
|  | T12-32ºC | 3.119 | 3119 |
|  | T24-32ºC | 2.98 | 2980 |
| ***P. acuta* cells** |  |  |  |
|  | T0- 25ºC | 1.197 | 1197 |
|  | T6- 25ºC | 1.199 | 1199 |
|  | T12- 25ºC | 1.242 | 1242 |
|  | T18- 25ºC | 1.239 | 1239 |
|  | T24- 25ºC | 1.158 | 1158 |
|  | T0-30ºC | 1.185 | 1185 |
|  | T6-30ºC | 1.146 | 1146 |
|  | T12-30ºC | 1.147 | 1147 |
|  | T18-30ºC | 1.127 | 1127 |
|  | T24-30ºC | 1.128 | 1128 |
| ***P. acuta* tissue** |  |  |  |
|  | T0-25ºC | 1.932 | 1932 |
|  | T12-25ºC | 2.001 | 2001 |
|  | T24-25ºC | 1.925 | 1925 |
|  | T12-30ºC | 1.949 | 1949 |
|  | T24-30ºC | 2.018 | 2018 |
