# Supplementary material for: Conserved HSP60 structure with lineage- and context-specific regulation in cnidarians
Source: Life Sci Alliance. 2026 Jun 24;9(9):e202503592. doi: 10.26508/lsa.202503592 (PMC13293977; doi:10.26508/lsa.202503592)
Supplement: Reviewer comments [file LSA-2025-03592_review_history.pdf]

Conserved HSP60 structure with lineage- and context-specific regulation in cnidarians

Saborni Chowdhury, Avery Kruger, and Liza Roger
DOI: https://doi.org/10.26508/lsa.202503592

Corresponding author(s): Liza Roger, Arizona State University

Review Timeline:

Submission Date:	2025-12-08
Editorial Decision:	2026-02-11
Revision Received:	2026-04-24
Editorial Decision:	2026-05-22
Revision Received:	2026-06-08
Accepted:	2026-06-16

Scientific Editor: Tim Fessenden

Transaction Report:

(Note: With the exception of the correction of typographical or spelling errors that could be a source of ambiguity, letters and reports are not edited. The original formatting of letters and referee reports may not be reflected in this compilation.)

February 11, 2026

Re: Life Science Alliance manuscript #LSA-2025-03592-T

Prof. Liza M. Roger
Arizona State University
School of Molecular Sciences
Walton Center For Planetary Health
777 E University Dr
TEMPE, Arizona 85281

Dear Dr. Roger,

Thank you for submitting your manuscript entitled "A conserved HSP60 architecture with lineage-and context-specific regulation reflects thermal resilience in cnidarians" to Life Science Alliance. The manuscript was assessed by expert reviewers, whose comments are appended to this letter. We appreciate your patience as we sought reviewer input.

As you will see, all reviewers remarked on the broad importance of these findings for understanding heat responses across cnidarians, and the intriguing observation that HSP60 sequence conservation is not reflected in its regulation during heat stress. However, reviewers were consistent in expressing concerns on methodology, quantification, and loading controls for western blot analysis that forms the core of this work. Reviewers 1 and 2 both requested a positive control for HSP60 detection in P acuta. Reviewer 1 also sought physiological observations and a clear justification for the temperatures used. In view of the consistent reviewer remarks, we feel that all reviewer comments must be fully addressed in a revised manuscript.

I would be happy to discuss the revision in more detail via email or phone/videoconferencing. Please let me know which option you prefer, if any.

To upload the revised version of your manuscript, please log in to your account: https://lsa.msubmit.net/cgi-bin/main.plex

You will be guided to complete the submission of your revised manuscript and to fill in all necessary information. Please get in touch in case you do not know or remember your login name.

The typical timeframe for revisions is three months. Please note that papers are generally considered through only one revision cycle, so strong support from the referees on the revised version is needed for acceptance.

While you are revising your manuscript, please also attend to the below editorial points to help expedite the publication of your manuscript. Please direct any editorial questions to the journal office. When submitting the revision, please include a letter addressing the reviewers' comments point by point.

Thank you for this interesting contribution to Life Science Alliance. We hope that the comments below will prove constructive as your work progresses, and we are looking forward to receiving your revised manuscript.

Sincerely,

Tim Fessenden
Scientific Editor
Life Science Alliance

A. THESE ITEMS ARE REQUIRED FOR REVISIONS

-- A letter addressing the reviewers' comments point by point.

-- An editable version of the final text (.DOC or .DOCX) is needed for copyediting (no PDFs).

-- High-resolution figure, supplementary figure and video files uploaded as individual files: See our detailed guidelines for preparing your production-ready images, https://www.life-science-alliance.org/authors

-- Summary blurb (enter in submission system): A short text summarizing in a single sentence the study (max. 200 characters

including spaces). This text is used in conjunction with the titles of papers, hence should be informative and complementary to the title and running title. It should describe the context and significance of the findings for a general readership; it should be written in the present tense and refer to the work in the third person. Author names should not be mentioned.

-- By submitting a revision, you attest that you are aware of our payment policies found here: https://www.life-science-alliance.org/copyright-license-fee

B. MANUSCRIPT ORGANIZATION AND FORMATTING:

Full guidelines are available on our Instructions for Authors page, https://www.life-science-alliance.org/authors

We encourage our authors to provide original source data, particularly uncropped/-processed electrophoretic blots and spreadsheets for the main figures of the manuscript. If you would like to add source data, we would welcome one PDF/Excel-file per figure for this information. These files will be linked as supplementary "Source Data" files.

IMPORTANT: It is Life Science Alliance policy that if requested, original data images must be made available. Failure to provide original images upon request will result in unavoidable delays in publication. Please ensure that you have access to all original microscopy and blot data images before submitting your revision.

Reviewer #1 (Comments to the Authors (Required)):

This manuscript presents an interesting comparative study of HSP60 regulation across three distinct cnidarian lineages (P. acuta, E. diaphana, and C. xamachana). The integration of physiological stress experiments with evolutionary analysis and AlphaFold structural modeling offers a valuable perspective on how conserved molecular architectures function in diverse biological contexts. The finding that P. acuta dissociated cells respond differently than intact tissue is particularly intriguing and contributes to the ongoing discussion about stress perception in corals.
The study addresses important questions regarding thermal resilience. However, to fully support the conclusions drawn, particularly regarding the "context-dependent" suppression of HSP60 in the coral P. acuta, the Western blot data requires some improvements.
Here are my specific comments:
1. The methods section states that 20 µg of total protein was loaded into each lane. However, the control beta-actin bands appear to vary considerably in intensity across the lanes, particularly in Figure 2A for the coral P. acuta tissue samples. This suggests that the total protein load might not have been consistent across all wells, which complicates the normalization process and may hence compromise the main argument of the manuscript about "context-dependent" regulation of HSP60.
2. The manuscript relies heavily on quantitative western blot analysis, yet there is only one setence in the Data Availability section mentioned "The western blot quantification was performed on ImageJ". Please expand the Methods section to include details on how the quantification was performed. And very importantly, quantitative western blot typically requires validation of the linear dynamic range of detection to ensure signals are not saturated. Did you verify that the band intensities (both target and loading control) fell within the linear range? This addition is crucial for supporting the "quantitative" aspect of the study.
3. The finding that HSP60 is undetectable in the coral P. acuta tissue samples is a key result. To better support this finding and definitively rule out potential sample-specific technical artifacts, it is necessary to run a positive control by spiking recombinant HSP60 into a P. acuta tissue lysate. If the antibody detects the spiked protein, it might be strong enough to support the conclusion that the lack of endogenous signal is a true biological phenomenon.
4. The authors link the HSP60 patterns to thermal resilience and bleaching susceptibility. To strengthen this link, it would be essential to include any physiological data collected during the experiment (e.g., PAM fluorometry/photosynthetic efficiency/visual bleaching scores). This would help confirm that the treatment induced a physiological stress response in the organisms, particularly for the coral P. acuta where no HSP60 was detected.
5. The study defines heat stress as +5 {degree sign}C above ambient aquaria temperature. While this is a standard delta, the baseline temperatures differ: 25 {degree sign}C for P. acuta, 22 {degree sign}C for E. diaphana, and 27 {degree sign}C for C. xamachana. Are these temperatures truly the physiological optimum for each specific holobiont strain? A +5 {degree sign}C shock might represent a different physiological severity for a tropical coral already near its thermal limit compared to a subtropical anemone. The manuscript would benefit from further justifications of why these specific baselines were chosen relative to the animals' thermal history.

Reviewer #2 (Comments to the Authors (Required)):

This manuscript investigates mitochondrial HSP60 regulation in response to acute thermal stress (+5 {degree sign}C) across three symbiotic cnidarians (Pocillopora acuta, Exaiptasia diaphana, and Cassiopea xamachana) using Western immunoblotting, supported by sequence, structural, and phylogenetic analyses. The integration of epitope conservation and AlphaFold-based structural mapping reinforces the evolutionary argument for antibody cross-reactivity, and the cross-taxa approach enhances

comparative insight. While the study presents a promising framework to probe stress responses in cnidarians, several important methodological and interpretive issues must be addressed before the manuscript can be considered for publication.

Major comments:
1. Lines 134-147: The text describes qualitative trends such as "gradual increase" or "no response," but lacks a transparent statistical framework to support these comparisons. Please clarify whether a two-way or mixed-effects ANOVA was used (e.g., species × temperature × time), whether interaction terms were included, and how assumptions and multiple testing were addressed. A dedicated "Statistical Analysis" subsection is recommended. Define biological vs. technical replicates, report exact p-values or confidence intervals, and include effect sizes where possible. For densitometry, detail the workflow used in ImageJ: band/ROI selection, background subtraction method, normalization strategy (e.g., relative to β-actin or total protein), and quality control steps (e.g., linearity validation, handling of weak/absent bands). This is critical for reproducibility and interpretation.
2. Lines 31-33; 136-137: The absence of detectable HSP60 in P. acuta tissue under control or heat stress is a key finding, but alternative explanations such as low expression, inefficient extraction, or poor antibody performance in this matrix must be ruled out. While recombinant HSP60 is used elsewhere (Fig. 2-3), it should be explicitly employed to define the assay's detection and quantification limits. This will help confirm that the absence of signal in P. acuta tissue lanes reflects biological absence/suppression rather than technical limitations.
3. Lines 135-136: β-actin is used as a loading control, but its expression can vary across taxa, tissue types, and under stress. Cross-species comparisons based on β-actin normalization alone may be unreliable. Please validate the stability of β-actin for each species and condition, or incorporate total protein normalization (e.g., Ponceau S or stain-free quantification). Ideally, present both normalization approaches to strengthen quantitative robustness.
4. Lines 374-386: The in vivo cross-species experiment is limited to three time points (T0, T12, T24) following a sudden +5 {degree sign}C increase. This relatively coarse resolution may miss key regulatory dynamics or transient responses. While practical constraints are understandable, please acknowledge this limitation and consider whether additional sampling time points (or data from dissociated cells) could refine temporal interpretation.
5. Lines 249-275; 314-316: The proposed interpretation that P. acuta's apparent lack of HSP60 induction is due to "loss of tissue-level signaling and buffering" or "suppression linked to bleaching susceptibility" is plausible but currently speculative. Please reframe this as a working hypothesis unless further evidence is provided. Supporting data such as HSP60 transcript levels, mitochondrial potential, ROS, ATP, or cell viability/apoptosis markers would help differentiate between true lack of induction versus technical or biological suppression.
6. Lines 861-862: The figure caption for Fig. 2 notes "n = 3, {plus minus}SE," but it is unclear whether these are biological replicates (e.g., distinct colonies/individuals) or technical replicates (e.g., repeated extractions or blots from the same sample). This distinction is essential for assessing statistical inference. Please clarify replicate definitions throughout and, where relevant, include tank/colony/sample identifiers in supplementary materials.

Minor comments:
1. Lines 31-33; 136-137: The terms "undetectable," "not detected," and "no response" are used interchangeably. Standardize terminology and clearly define what is meant by "undetectable" (i.e., below detection threshold of the assay, not biologically absent).
2. Fig. 2-3 captions, Lines 859-870: Include the normalization formula used (e.g., HSP60/β-actin or HSP60/total protein), background subtraction method, and whether values are averaged across technical replicates. This improves transparency and interpretability.
3. Lines 442-449; 472-473: The manuscript mentions "MSA using pairwise alignment" and later "Clustal Omega." Please clarify which aligner was used for each step (MSA, phylogenetics, epitope conservation), with version numbers and alignment parameters.
4. Lines 182-184; 465-469: The manuscript refers to both "AlphaFold3" and "AlphaFold Version 4." Please ensure versioning is accurate and consistent. Indicate the database or platform used (e.g., AlphaFold DB, ColabFold) and provide accession identifiers or sequence inputs.
5. Lines 510-518: While OSF, Protocols.io, and sequence sources are cited, ensure that all links are publicly accessible and include accession IDs where applicable. For protocol repositories, confirm that reviewer access is enabled or consider including key workflows in the supplementary material.

Reviewer #3 (Comments to the Authors (Required)):

The manuscript presents a well-developed and coherent description of the research problem, supported by appropriate and up-to-date references. The objective is clearly stated, and the study includes three different species of symbiotic cnidarians, which strengthens the comparative scope of the work. The results regarding HSP60 expression in the three species are interesting and contribute relevant information to the field. However, some of the figures do not meet a high standard of image quality (Figure 2 B), likely due to methodological issues related to the electrophoresis technique; in my view, this limitation compromise the validity of the results or their overall interpretation, but improving the image quality would enhance the clarity and impact of the presentation. If this is not possible, I suggest removing this species from the study or explaining more clearly how the low quality image and the normalization in the bar graph should be interpreted.

The work exposes a clear disconnect between the deep conservation of HSP60 and its uneven thermal regulation across cnidarians. The contrasting patterns in Pocillopora, Exaiptasia, and Cassiopea underscore how lineage and biological context shape mitochondrial proteostasis. Yet the reliance on a single antibody also limits interpretation: isoform-specific expression-detectable at the transcript level (RNA) but invisible to immunoblotting, may underlie some of the observed discrepancies. These findings caution against assuming uniform stress responses and highlight the need for multi-chaperone, multi-omic approaches to understand thermal tolerance and bleaching vulnerability. However, the authors indicate the limitations of the work on line 327.

Although the methods section describes how the membrane signal was captured and quantified, the result associated with the membrane image itself is not addressed in the results section.It would be helpful to clarify how the quantification software identifies the area to be measured in relation to the figure showing the normalized intensity bar plots. Additionally, were the three replicates associated with the same membrane image, or does each bar correspond to an independent image/replicate.

I suggest including the case of Hydra oligactis in the Introduction. Please review the following papers:
Bosch TC, Krylow SM, Bode HR, Steele RE. Thermotolerance and synthesis of heat shock proteins: these responses are present in Hydra attenuata but absent in Hydra oligactis. Proc Natl Acad Sci U S A. 1988 Nov;85(21):7927-31. doi: 10.1073/pnas.85.21.7927. PMID: 3186697; PMCID: PMC282326.
Clark MS, Fraser KP, Peck LS. Antarctic marine molluscs do have an HSP70 heat shock response. Cell Stress Chaperones. 2008 Spring;13(1):39-49. doi: 10.1007/s12192-008-0014-8. Epub 2008 Feb 7. PMID: 18347940; PMCID: PMC2666218.

All text in blue font has been newly added per the reviewer's comment. This is to help distinguish new text from old.

Comments: 19/19 addressed

Reviewer-1:

Rev.1 Comment 1: The method section states that 20 µg of total protein was loaded into each lane. However, the control beta-actin bands appear to vary considerably in intensity across the lanes, particularly in Figure 2A for the coral P. acuta tissue samples. This suggests that the protein load might not have been consistent across all wells, which complicates the normalization process and may hence compromise the main argument of the manuscript about "context-dependent" regulation of HSP60

Reply to Rev.1 Comment 1: We thank the reviewer for pointing this out. We agree that the variability in β-actin band intensity across lanes, particularly in the *P. acuta* tissue samples in Figure 2A, complicates the use of β-actin as the normalization control. To address this concern, we normalized HSP60 band intensity to total protein and incorporated this analysis into the revised manuscript.
Specifically, we included a boxplot showing relative HSP60 densitometry normalized to total protein for all three species. We also added this data to the Results and Supplementary Information and revised the Results and Discussion sections accordingly. This data provides an additional normalization framework for evaluating HSP60 patterns and strengthens the interpretation of context-dependent regulation. We note that normalization methods yielded partially different statistical outcomes; therefore, results are interpreted conservatively and presented using both approaches.

Lines 133-137:

To account for variability in β-actin band intensity across lanes, we additionally normalized HSP60 band intensity to total protein. This analysis provided an independent framework for evaluating HSP60 expression patterns across species and experimental conditions. Relative HSP60 densitometry normalized to total protein is presented in Fig.2.

Lines 338-342:

β-actin band intensity varied across certain samples, especially in *P. acuta* tissue, making normalization to the loading control less reliable for analysis purposes. This variation may reflect the effects of heat-stress on actin expression (47-48). We therefore used total protein normalization as an additional approach to interpret relative HSP60 band intensity

Rev.1 Comment 2: The manuscript relies heavily on quantitative Western blot analysis, yet there is only one sentence in the Data availability section mentioned: " The western blot quantification was performed on ImageJ". Please expand the method section to include details on how the quantification was performed. And very importantly, a quantitative western blot typically requires validation of the linear dynamic range of detection to ensure signals are not saturated. Did you verify that the band intensities (both target and loading control) fell within the linear range? This addition is crucial for supporting the "quantitative" aspect of the study

Reply to Rev.1 Comment 2: We appreciate the reviewer's thoughtful comment. We have revised the manuscript accordingly. The Methods section now includes detailed information on image acquisition, ROI selection, background subtraction, normalization, and treatment of weak or absent bands. We also revised the manuscript to present the western blot data as relative band intensities rather than as fully quantitative values, and we now note the lack of formal linear dynamic range validation as a limitation in the Discussion. During experimental optimization, we examined HSP60 and β-actin expression across a range of heat-stress durations and temperatures. These exploratory conditions were not used for quantitative analysis and do not substitute for formal validation of linear dynamic range. Protein loading was standardized by Bradford assay and additionally assessed by SDS-PAGE before immunoblot analysis. These clarifications have now been incorporated into the revised manuscript.

Lines 1017-1018:
Western blot signals were quantified by densitometry and reported as relative band intensity, normalized to β-actin and total protein.

Lines 543-554:
Band intensities were quantified using ImageJ/Fiji. Original chemiluminescent blot images were opened without contrast enhancement for measurement. A uniform rectangular region of interest (ROI) was drawn around each band, and the same ROI size was maintained for all lanes on a given blot. Integrated density was measured for each target and loading-control band. To correct for the nonspecific signal, the background was measured from an adjacent membrane region lacking visible bands and subtracted from the raw band intensity. The corrected HSP60 signal for each lane was normalized to the corrected β-actin signal from the same lane. The resulting normalized values were used as relative densitometric measurements. Since a formal dilution-series experiment was not performed, the linear dynamic range of detection for HSP60 and β-actin was not explicitly validated here.

Rev.1 Comment 3: The finding that HSP60 is undetectable in the coral P.acuta tissue sample is a key result. To better support this finding and definitively rule out sample–specific technical artifacts, it is necessary to run a positive control by spiking recombinant HSP60 into a P. acuta tissue lysate. If the antibody detects the spiked protein, it might be strong enough to support the conclusion that the lack of endogenous signal is a true biological phenomenon.

Reply to Rev.1 Comment 3: We thank the reviewer for this thoughtful and constructive suggestion. The experiment suggested by the reviewer was not performed as part of the present study. We have revised the manuscript to present a more accurate interpretation of this result. Specifically, we now describe the finding as an absence of detectable HSP60 signal in *P. acuta* under the conditions tested, rather than as definitive evidence of biological absence. Given the absence of a reproducible detectable signal and the lack of matrix-specific validation, we interpret this result conservatively as reflecting detection limits under the present experimental conditions rather than definitive protein absence. We have also added text to the Discussion acknowledging that recombinant HSP60 spike-in experiments would be an important next step for validating antibody performance in *P. acuta* lysates. In parallel, we are exploring complementary strategies to establish a biologically relevant positive control for HSP60 detection in cnidarian systems, including pharmacological treatments designed to induce HSP60 expression. Since an inducible positive-control framework has not yet been established in the cnidarian system, this is an important direction for future method development.

Lines 373-375:
Future studies should incorporate recombinant HSP60 spike-in assays as well as pharmacological induction approaches to establish appropriate positive controls for HSP60 detection in cnidarian tissues.

Lines 138-139:
In *P. acuta* tissue lysates, no detectable HSP60 signal was observed by western blot under the experimental conditions.

Rev.1 Comment 4: The authors link the HSP60 patterns to thermal resilience and bleaching susceptibility. To strengthen this link, it would be essential to include any physiological data collected during the experiment (e.g., PAM fluorometry, photosynthetic efficiency, visual bleaching scores). This would help confirm that the treatment induced a physiological stress response in the organisms, particularly for the coral *P.acuta* where no HSP60 was detected.

Reply to Rev.1 Comment 4: We acknowledge that physiological measurements such as PAM fluorometry, photosynthetic efficiency, and visual bleaching scores would provide valuable complementary context. These experiments were not included in the present study, as our primary aim was to characterize the acute cellular heat-shock response in symbiotic cnidarians under short-term thermal stress. Our experiments used a 24 h exposure window to target early molecular responses, prior to the onset of bleaching-associated physiological changes. Although these organisms contain photosynthetic algal symbionts, a detailed assessment of symbiont photophysiology was not part of our experimental aims; this work focused specifically on host-associated cellular stress responses under acute heat stress.
We appreciate the reviewer raising this important point. We have therefore revised the manuscript to clearly acknowledge the limited basis for drawing direct links between HSP60 expression patterns and thermal resilience or bleaching susceptibility in the absence of physiological measurements. This remains an important direction, and we hope the current findings will motivate future work combining cellular and physiological assessments.

Rev.1 Comment 5: This study defines heat stress as +5 ºC above ambient aquaria temperature. While this is the standard delta, the baseline temperatures differ: 25 ºC for P.acuta, 22ºC for E. diaphana and 27ºC for C.xamachana. Are these temperatures truly the physiological optimum for each specific holobiont strain? A +5ºC shock might represent a different physiological severity for a tropical coral already near its thermal limit compared to a subtropical anemone. The manuscript would benefit from further justifications of why these specific baselines were chosen relative to the animal's thermal history.

Reply to Rev.1 Comment 5: We thank the reviewer for this insightful comment. We have revised the manuscript to clarify the rationale of our experimental design. Our goal in this study was not to expose all species to an ecologically equivalent heat-stressed condition. We did not design our experiment so that the chosen baseline temperatures correspond to the precise physiological optima of each holobiont strain in nature. All organisms were maintained under controlled laboratory conditions and not collected directly from the field.

Hence, **the baseline temperatures were selected according to established husbandry protocols optimized for each species– *P.acuta* (corals), *C.xamachana* (upside-down jellyfish), and *E. diaphana* (sea anemones)**. The +5 °C treatment was then applied as a standardized thermal exposure relative to each organism's laboratory-maintained ambient growth temperature, allowing consistent comparison of acute HSP60 responses across taxa, under controlled experimental conditions. We have clarified this rationale in the Discussion that the imposed heat treatment was designed as a standardized laboratory heat-stress condition relative to the baseline husbandry conditions and should not be interpreted as a direct simulation of equivalent physiological stress across taxa.

Lines 310-317:
To ensure experimental consistency across taxa, we applied a standardized increase of +5 °C relative to the baseline husbandry temperature used for each organism. We acknowledge that this experimental increase was not intended to reproduce the exact thermal conditions of each species' natural habitat. However, because all organisms were maintained under controlled laboratory conditions rather than sampled directly from the field, their baseline temperatures were based on established husbandry practices tailored to each taxonomic group represented in the study.

Reviewer-2:
Major comments

Rev.2 Major Comment 1: Lines 134-147: The text describes qualitative trends such as "gradual increase" or "no response," but lacks a transparent statistical framework to support these comparisons. Please clarify whether a two-way or mixed-effects ANOVA was used (e.g., species × temperature × time), whether interaction terms were included, and how assumptions and multiple testing were addressed. A dedicated "Statistical Analysis" subsection is recommended. Define biological vs. technical replicates, report exact p-values or confidence intervals, and include effect sizes where possible. For densitometry, detail the workflow used in ImageJ: band/ROI selection, background subtraction method, normalization strategy (e.g., relative to β-actin or total protein), and quality control steps (e.g., linearity validation, handling of weak/absent bands). This is critical for reproducibility and interpretation.

Reply to Rev.2 Major Comment 1: We thank the reviewer for this valuable insight. We agree that the original wording in the Results relied too heavily on qualitative descriptions and did not explain clearly enough how the densitometry data were evaluated statistically. We have revised the manuscript accordingly. Please see the revised text below.

A dedicated Statistical Analysis subsection has now been added to the Methods. Since the experimental design differed among species and sample types, each experimental system was analyzed separately. For datasets with measurable signal and sufficient replication, we used two-way ANOVA with temperature, time, and the temperature : time interaction included in the model. Technical replicates, where available, were averaged prior to analysis, and all reported n values refer to biological replicates.

We also clarified how ANOVA assumptions were evaluated. Specifically, model assumptions were assessed by inspecting residuals and using Shapiro–Wilk tests for normality and Levene's tests for homogeneity of variance. These checks did not indicate major violations for the datasets analyzed by ANOVA. Exact p-values are now reported in the revised Results. The Results and Discussion sections have been revised accordingly

to align the interpretation with the statistical support.

Lines 556-579:

Statistical analysis

Each experimental system was analyzed separately to reflect differences in study design among species and sample types, rather than combining all datasets into a single cross-species model. For datasets with measurable signal and replication, normalized HSP60 values were analyzed using two-way ANOVA with temperature, time, and the temperature: time interaction as fixed effects. For *E. diaphana* and *C. xamachana*, only time points common to both temperature treatments were included. For each analysis, n refers to independent biological replicates (n = 3 per condition), where each replicate corresponds to a separate organism or independent cell preparation maintained in an individual well. Technical replicates were averaged prior to analysis. Two-way ANOVA models included temperature (2 levels) and time (3 or 5 levels, depending on the dataset), and the degrees of freedom reflect the number of factor levels and total independent observations included in each model. Model assumptions were evaluated by examining residuals and by applying Shapiro–Wilk tests for normality and Levene's tests for homogeneity of variance. Exact *p*-values are reported where relevant, and statistical significance was set at $p < 0.05$. For *P. acuta* tissue samples, normalized HSP60 values were uniformly zero across all conditions. Because there was no measurable variation in the response variable, formal ANOVA was not informative for this dataset, and the results were interpreted descriptively. Each experimental system was analyzed independently with predefined comparisons, therefore no formal correction for multiple testing was applied; results are interpreted within each system rather than across all analyses. Given the small sample size (n = 3), effect sizes were not emphasized, and results are reported primarily using F-statistics and p-values.

Lines 185-218:

Statistical analysis of the two-way ANOVA test of HSP60 across cnidarian systems

Each analysis was performed on independent biological replicates (n = 3 per condition), and sample sizes differed across datasets depending on the number of time points included.

In *E. diaphana*, two-way ANOVA of β-actin-normalized HSP60 intensity revealed significant main effects of temperature ($F_{1,8} = 5.405$, $p = 0.0486$) and time ($F_{1,8} = 5.721$, $p = 0.0437$), while the temperature × time interaction was not significant ($F_{1,8} = 0.844$, $p = 0.3852$). In contrast, total-protein-normalized HSP60 intensity did not show significant effects of temperature ($F_{1,8} = 4.311$, $p = 0.0715$), time ($F_{1,8} = 1.232$, $p = 0.2992$), or their interaction ($F_{1,8} = 0.606$, $p = 0.4588$). Together, these results suggest a temperature-associated pattern in *E. diaphana*, with stronger statistical support under β-actin normalization than under total-protein normalization (Fig S6B).

In *C. xamachana*, β-actin-normalized HSP60 intensity showed a significant effect of temperature ($F_{1,8} = 16.129$, $p = 0.00386$), whereas time ($F_{1,8} = 0.003$, $p = 0.96131$) and the temperature × time interaction ($F_{1,8} = 1.456$, $p = 0.26200$) were not significant. Under total-protein normalization, the temperature effect did not reach significance but approached it ($F_{1,8} = 4.685$, $p = 0.0623$), while time ($F_{1,8} = 0.985$, $p = 0.3501$) and the interaction ($F_{1,8} = 0.015$, $p = 0.9055$) remained non-significant. Results suggest that HSP60 expression in *C. xamachana* was more strongly associated with temperature than with

exposure duration during the sampled period (Fig S6C).

In dissociated *P. acuta* cells, two-way ANOVA did not detect significant effects of temperature ($F_{1,20}$ = 0.019, p = 0.893), time ($F_{4,20}$= 0.228, p = 0.920), or their interaction (F4,20=0.428, p = 0.787) when HSP60 was normalized to β-actin. The same pattern was observed with total-protein normalization, where temperature ($F_{1,20}$ = 1.103, p = 0.306), time ($F_{4,20}$ = 0.433, p = 0.783), and the temperature × time interaction ($F_{4,20}$= 0.519, p = 0.723) were all non-significant. Thus, although HSP60 signal was detectable in dissociated *P. acuta* cells, it was not significantly regulated across the tested conditions (Fig S6A).

For the datasets analyzed by ANOVA, inspection of residuals, along with Shapiro–Wilk and Levene's tests, did not indicate major violations of model assumptions.

Lines 332-338:

Additionally, the statistical analyses indicate that HSP60 regulation was not uniform across the systems examined. The strongest statistical support was observed in *E. diaphana*, while *C. xamachana* showed a temperature-associated pattern. In *P. acuta*, HSP60 was detectable in dissociated cells but showed no significant changes across conditions, and was not detected in tissue samples. These findings support a context-dependent interpretation of HSP60 expression across cnidarian systems.

Lines 544-555:

Band intensities were quantified using ImageJ/Fiji. Original chemiluminescent blot images were opened without contrast enhancement for measurement. A uniform rectangular region of interest (ROI) was drawn around each band, and the same ROI size was maintained for all lanes on a given blot. Integrated density was measured for each target and loading-control band. To correct for the nonspecific signal, the background was measured from an adjacent membrane region lacking visible bands and subtracted from the raw band intensity. The corrected HSP60 signal for each lane was normalized to the corrected β-actin signal from the same lane. The resulting normalized values were used as relative densitometric measurements. Since a formal dilution-series experiment was not performed, the linear dynamic range of detection for HSP60 and β-actin was not explicitly validated here.

Rev.2 Major Comment 2: Lines 31-33; 136-137: The absence of detectable HSP60 in P. acuta tissue under control or heat stress is a key finding, but alternative explanations such as low expression, inefficient extraction, or poor antibody performance in this matrix must be ruled out. While recombinant HSP60 is used elsewhere (Fig. 2-3), it should be explicitly employed to define the assay's detection and quantification limits. This will help confirm that the absence of signal in P. acuta tissue lanes reflects biological absence/suppression rather than technical limitations.

Reply to Rev.2 Major Comment 2: We thank the reviewer for this important comment. We agree that the absence of detectable HSP60 in *P. acuta* tissue requires careful evaluation of possible technical explanations. In response, we clarified in the revised manuscript that recombinant HSP60 was used as a positive control in the relevant western blot assays, including those containing *P. acuta* tissue samples (Figures 2-3). This demonstrates that the antibody detected HSP60 in *P. acuta* tissue lysates under our assay conditions as well. We also quantified total protein prior to loading by Bradford assay and

assessed multiple primary antibody dilutions during assay optimization (**S9 and S10**). Although these controls support the assay's technical performance, we acknowledge that we did not perform a recombinant dilution series to define the assay's detection and quantification limits. We have therefore revised the manuscript to describe this result conservatively as HSP60 not being detected in *P. acuta* tissue under the conditions tested, and we now explicitly note this limitation in the Discussion.

Lines 524-526:
Recombinant HSP60 was included as a positive control in the relevant western blot assays, including those performed with *P. acuta* tissue lysates. Total protein concentration was measured prior to loading using the Bradford assay.

Lines 155-162:
Detection of recombinant HSP60 on the corresponding blots confirmed antibody reactivity under the assay conditions used. However, since recombinant HSP60 was not spiked into the cell lysates and a formal detection limit was not established for this matrix, we cannot fully exclude the possibility that low abundance or matrix-specific effects contributed to the lack of detectable signal in the sample. Accordingly, these findings are interpreted conservatively as variable or below detection-level expression rather than definitive absence.

Rev.2 Major Comment 3: Lines 135-136: β-actin is used as a loading control, but its expression can vary across taxa, tissue types, and under stress. Cross-species comparisons based on β-actin normalization alone may be unreliable. Please validate the stability of β-actin for each species and condition, or incorporate total protein normalization (e.g., Ponceau S or stain-free quantification). Ideally, present both normalization approaches to strengthen quantitative robustness.

Reply to Rev.2 Major Comment 3: We thank the reviewer for this important comment. We agree that β-actin expression may vary across taxa, tissue types, and stress conditions, which can limit its suitability as a sole normalization reference, particularly in cross-species comparisons. To address this concern, we have revised the manuscript to incorporate an additional normalization approach based on total protein. In addition to the **HSP60/β-actin** analysis, we now added **HSP60/total protein** normalization as a complementary analysis and included these data in Figures 2-3. We have also revised the relevant sections of the manuscript to clarify that interpretation of the western blot data does not rely exclusively on β-actin normalization. This additional analysis strengthens the robustness of the relative HSP60 comparisons across species and experimental conditions. We have also revised the text throughout the manuscript to clarify that western blot results are interpreted using both β-actin and total protein normalization, rather than relying solely on β-actin. We acknowledge this limitation and present total-protein normalization as an additional analytical framework to support the interpretation of the HSP60 expression patterns. We have revised the Methods, Results, Discussion, and figure captions to clarify the normalization strategy and to incorporate total-protein normalization as a complementary analytical approach.

Lines 133-137:

To account for variability in β-actin band intensity across lanes, we additionally normalized HSP60 band intensity to total protein. This analysis provided an independent framework

for evaluating HSP60 expression patterns across species and experimental conditions. Relative HSP60 densitometry normalized to total protein is presented in Fig.2.

Rev.2 Major Comment 4: Lines 374-386: The in vivo cross-species experiment is limited to three time points (T0, T12, T24) following a sudden +5 °C increase. This relatively coarse resolution may miss key regulatory dynamics or transient responses. While practical constraints are understandable, please acknowledge this limitation and consider whether additional sampling time points (or data from dissociated cells) could refine temporal interpretation.

Reply to Rev.2 Major Comment 4: We thank the reviewer for this important comment. We acknowledge that the *in vivo* cross-species experiment with the sampling at T0, T12, and T24 following an acute +5 °C temperature increase, provides limited temporal resolution and may not capture transient or rapidly changing HSP60 responses. We agree that additional sampling points would further refine the interpretation of the temporal dynamics. Our aim in this study was to apply a standardized *in vivo* sampling framework across species under controlled laboratory conditions in order to compare acute HSP60 responses. Within these practical constraints, T0, T12, and T24 were selected as common time points for all taxa. We have revised the Discussion to explicitly acknowledge that this design may have missed transient or intermediate protein expression occurring outside the sampled intervals. To address this limitation more clearly, we now also refer to the *P. acuta* dissociated-cell experiments as complementary evidence that HSP60 expression can vary across biological contexts and may exhibit patterns not fully captured in the *in vivo* time course. In addition, we have added a summary table (S9) that compiles all experiments, including experimental conditions, sampling intervals, and whether HSP60 was detected in each system. This addition improves transparency and helps contextualize the temporal findings.

Lines 318-332:

To standardize the *in vivo* comparison across taxa, sampling was performed at three shared time points (T0, T12, and T24) following an acute +5 °C increase above each species' laboratory-maintained baseline temperature. Although this design enabled direct comparison across systems, the relatively coarse temporal resolution may have missed transient or rapidly changing HSP60 responses that occurred between sampling intervals. Within these constraints, *P. acuta* tissue lysates did not show reproducible detectable HSP60 expression, *E. diaphana* showed a progressive increase in HSP60 signal over time under heat stress, and *C. xamachana* maintained constitutive expression with temperature-dependent variation in signal intensity. In contrast, dissociated *P. acuta* cells exhibited variable HSP60 expression under both control and heat-stress conditions, further suggesting that HSP60 dynamics may depend on both temporal resolution and biological context. SI 9 summarizes HSP60 detection patterns across all experiments for better interpretation of these results.

Rev.2 Major Comment 5: Lines 249-275; 314-316: The proposed interpretation that P. acuta's apparent lack of HSP60 induction is due to "loss of tissue-level signaling and buffering" or "suppression linked to bleaching susceptibility" is plausible but currently speculative. Please reframe this as a working hypothesis unless further evidence is provided. Supporting data such as HSP60 transcript levels, mitochondrial potential, ROS, ATP, or cell viability/apoptosis markers would help differentiate between true lack of

induction versus technical or biological suppression.
**Reply to Rev.2 Major Comment 5:** Thank you very much for bringing this to our attention. Please find the newly revised text below.<br><br>Lines 353-358: While further research is needed in *in vitro* cnidarian cellular biology, we propose that these differences in *P. acuta* HSP60 expression are best explained by biological rather than technical factors. In dissociated cell samples, tissue-level signaling, metabolic coupling, and intracellular feedback loops are disrupted, which we hypothesize leads to altered stress perception and loss of physiological buffering within the organism (49-51).<br><br>Line 356-374: Consequently, equivalent thermal challenges may trigger adaptive HSP60 induction in isolated cells compared to intact tissue. Comparable phenomena are observed in mammalian systems, where cell detachment affects HSF1 activation and alters HSP60 and HSP70 induction due to disrupted integrin signaling and mitochondrial instability (52-55). This cross-system parallel suggests that HSP60 regulation is fundamentally dependent on integrated physiological context, tissue organization, and taxa rather than temperature alone, supporting a hierarchical model of stress regulation that is altogether context-, lineage-, and temperature-dependent. For the thermally sensitive *P. acuta*, we hypothesize that the absence of detectable HSP60 induction in intact tissue fragments suggests that physiological buffering or successful tissue integrity signaling may suppress the immediate initiation of mitochondrial stress and secondary stress pathways (including those involving HSP60's roles in apoptosis and immune modulation). This suppression, however, prevents the deployment of a critical molecular defense mechanism necessary to manage proteostatic demand, thereby contributing to the species' thermal sensitivity and increased bleaching susceptibility. Future studies should incorporate recombinant HSP60 spike-in assays as well as pharmacological induction approaches to establish appropriate positive controls for HSP60 detection in cnidarian tissues.

**Rev.2 Major Comment 6:** Lines 861-862: The figure caption for Fig. 2 notes "n = 3, ±SE," but it is unclear whether these are biological replicates (e.g., distinct colonies/individuals) or technical replicates (e.g., repeated extractions or blots from the same sample). This distinction is essential for assessing statistical inference. Please clarify replicate definitions throughout and, where relevant, include tank/colony/sample identifiers in supplementary materials.
**Reply to Rev.2 Major Comment 6:**<br>We thank the reviewer for this helpful observation. We have revised the Methods section and figure legends to clarify that **n = 3 denotes biological replicates** throughout the manuscript. In the *P. acuta* fragment experiments, each biological replicate corresponded to a sample obtained from a distinct colony, rather than repeated sampling of the same colony or technical repetition of the same extract or blot. Different colonies were used at each time point to avoid confounding effects associated with repeated tissue sampling. A total of 18 colonies were used in this experiment, including 9 under control conditions and 9 under heat treatment.

For the *P. acuta* cell-based experiments, **n = 3** likewise refers to biological replicates. Each replicate consisted of an independent cell dissociation prepared from a different fragment/nubbin and standardized to **1 × 10^6 cells mL^{-1}** at each sampling point.
For *E. diaphana*, each biological replicate consisted of a pooled sample of 6 independent individuals collected at a given time point. This yielded 3 biological replicates for western blot analysis. A total of 30 anemones were used per experiment, and 90 anemones across all experiments.
For *C. xamachana*, each biological replicate consisted of one independent medusa collected at a given time point. A total of 5 medusae were used per experiment, and 15 medusae across all experiments. We have revised the method section with the following lines.

Supplementary Information-S2: Throughout the study, n=3 denotes biological replication. In *P. acuta* fragment experiments, each replicate was derived from a different colony. In the corresponding cell-based experiments, each replicate consisted of an independent cell preparation from a separate coral fragment (nubbin) and was normalized to 1 × 10^6 cells mL^{-1} at each sampling point. For *E. diaphana*, replicates were based on pooled samples from 6 independent individuals, while for *C. xamachana*, each replicate represented a single, independently collected medusa.

Minor comments:

Rev. 2 Minor Comment 1: Lines 31-33; 136-137: The terms "undetectable," "not detected," and "no response" are used interchangeably. Standardize terminology and clearly define what is meant by"undetectable" (i.e., below the detection threshold of the assay, not biologically absent).

Reply to Rev.2 Minor Comment 1: We thank the reviewer for this helpful comment. We agree that the terms “undetectable,” “not detected,” and “no response” should be used consistently and defined more clearly. We have revised the manuscript throughout to standardize this terminology. Specifically, we now use **“not detected”** to indicate that the HSP60 signal was **below the western blot assay's detection threshold.**

The relevant statements in the Abstract and Results have been revised accordingly to ensure consistent use of this definition throughout the manuscript. During assay optimization for *P. acuta*, we also assessed multiple primary antibody dilutions (1:250, 1:500, and 1:1000) and included recombinant HSP60 as a positive control on the blots. These observations indicate that the absence of an endogenous signal in *P. acuta* cannot be attributed exclusively to antibody failure. Nonetheless, to maintain a conservative interpretation, we now state that HSP60 was **not detected** under the conditions tested in this study. Please find the following revised lines:

Lines 35-41: We evaluated HSP60 expression in *Pocillopora acuta* (hard coral), *Exaiptasia diaphana* (sea anemone), and *Cassiopea xamachana* (upside-down jellyfish) using immunoblotting. In *P. acuta*, HSP60 was not detected at the fragment level under either control (25 ºC) or heat-stress (30 ºC). In contrast, isolated cells showed transient HSP60 expression under both temperature conditions, indicating context-dependent regulation in coral.

Lines 162-169: In addition to β-actin-based normalization, in *P. acuta* cells, HSP60 was

not detected under either control (25 °C) or heat-stress (30 °C) conditions (Fig. 2A), ("not detected" defined as a signal below the detection threshold of the western blot assay). In *E. diaphana*, transient HSP60 expression was observed under control conditions (22 °C) and increased at 12 h and 24 h during heat stress (27 °C; Fig. 2B). In *C. xamachana*, HSP60 was detected constitutively across all time points under both ambient (27 °C) and heat-stress (32 °C) conditions (Fig. 2C), with signal intensity increasing over time at elevated temperature.

Rev. 2 Minor Comment 2: Fig. 2-3 captions, Lines 859-870: Include the normalization formula used (e.g., HSP60/ß-actin or HSP60/total protein), background subtraction method, and whether values are averaged across technical replicates. This improves transparency and interpretability.

Reply to Rev. 2 Minor Comment 2: We thank the reviewer for this helpful comment. We have revised the Figure 2–3 captions and the Methods section to clarify the densitometric analysis in greater detail. Specifically, the primary normalization shown in Figures 2–3 is **HSP60/β-actin**. In response to the related concern regarding variability in the loading-control signal, we also performed **HSP60/total protein** normalization and have included the corresponding analyses in Figures 2-3.

We have further clarified the background-correction procedure and now specify that densitometric values were obtained following background subtraction prior to normalization. In addition, the revised captions indicate whether the plotted values represent averages across technical replicates. These revisions were made to improve the transparency and interpretability of the Western blot analysis. Please find the following revised texts:

Lines 1029-1034: Representative western blots and corresponding densitometric quantification of HSP60 under control and heat-stress conditions. Densitometric measurements were performed in ImageJ after local background subtraction, and the HSP60 signal was normalized to the corresponding β-actin (B) and total protein signal (C) . Data are presented as relative band intensity and represent the mean of technical replicates for each biological replicate (n=3, mean ± SE).

Rev. 2 Minor Comment 3: Lines 442-449; 472-473: The manuscript mentions "MSA using pairwise alignment" and later "Clustal Omega." Please clarify which aligner was used for each step (MSA, phylogenetics, epitope conservation), with version numbers and alignment parameters.

Reply to Rev. 2 Minor Comment 3: Thank you for bringing this point to our attention. Please find the additional information in the revised text below.

Lines 583-597: *Multiple Sequence Alignment*

To assess evolutionary conservation and sequence homology of HSP60 across representative cnidarian taxa, multiple sequence alignment (MSA) using pairwise alignment was performed on NCBI BLASTp using HSP60 amino acid sequences derived from *P. acuta*, *E. diaphana*, and *C. xamachana*, alongside the canonical human HSP60 (HSPD1, UniProt ID: P10809) as reference. The candidate coral (*P. acuta*) sequences were obtained from Vidal-Dupiol *et al.* (92), *C. xamachana* sequences from the Joint Genome Institute (https://jgi.doe.gov/), and *E. diaphana* sequences from ReefGenomics (95-96). The results from this alignment provided orthologs for *P. acuta* (596 aa), *E. diaphana* (586 aa), and *C. xamachana* (583 aa). The pairwise alignments show a highly conserved amino acid sequence within the C-terminal region (alignment ranges 391-434). The identities for these candidate HSP60 sequences are 85% (*P. acuta* and *E. diaphana*) and 89% (*C. xamachana*), with 100% positives and no gaps present (E-values between 8×10^{-20} and 6×10^{-20}).

Lines 622-632: Conserved regions corresponding to the human HSP60 epitope (amino acid 383-419) were identified using Clustal Omega multiple sequence alignment on NCBI BLASTp (94). Pairwise alignments using the query 37 amino acid epitope from *P. acuta* show strong conservation in *E. diaphana* (35/37 identities; 95% identity; 100% positives; 0 gaps; E-value 8×10^{-24}) and *C. xamachana* (34/37 identities; 92% identity; 100% positives; 0 gaps; E-value 2×10^{-2}). Homologous residues were mapped to each cnidarian HSP60 sequence (positions 398–434 in *P. acuta*, 393–429 in *E. diaphana,* and 391–427 in *C. xamachana*) and highlighted on their respective AlphaFold-predicted tertiary structures. These residues were rendered in cartoon representations (cyan) to evaluate spatial conservation, solvent exposure, and structural context of the conserved epitope within the chaperonin architecture.

Rev. 2 Minor Comment 4: Lines 182-184; 465-469: The manuscript refers to both "AlphaFold3" and "AlphaFold Version 4." Please ensure versioning is accurate and consistent. Indicate the database or platform used (e.g., AlphaFold DB, ColabFold) and provide accession identifiers or sequence inputs.

Reply to Rev. 2 Minor Comment 4: Thank you very much for pointing out this discrepancy. Please find the corrected versions below.

Lines 257-262: To connect sequence conservation to structural context, we predicted HSP60 tertiary structures for *P. acuta*, *E. diaphana*, and *C. xamachana* using AlphaFold3 and mapped the 37-residue antigenic motif identified by pairwise alignments to human HSP60 (HSPD1, Fig.5) (32). AlphaFold3 consistently recovered the canonical domain organization across all three cnidarians, placing the conserved epitope in an equivalent position on the equatorial domain surface (Fig.5).

Lines 614-621: *AlphaFold3 structural prediction and epitope mapping*

Predicted tertiary structures of HSP60 homologs from *P. acuta* (accession ID: TCONS_00030188), *C. xamachana* (accession ID: Casxa1|9735), and *E. diaphana* (accession ID: AIPGENE15267) were obtained from the AlphaFold Protein Structure

database (DeepMind and EMBL). For each species, the highest-ranked model was downloaded (AlphaFold Version 3) in CIF format and visualized in UCSF ChimeraX (Version 1.10.1). Per-residue confidence scores were derived from the predicted local distance difference test (pLDDT) values.

Rev. 2 Minor Comment 5: Lines 510-518: While OSF, Protocols.io, and sequence sources are cited, ensure that all links are publicly accessible and include accession IDs where applicable. For protocol repositories, confirm that reviewer access is enabled or consider including key workflows in the supplementary material.

Reply to Rev. 2 Minor Comment 5: Thank you for bringing this to our attention. While we ensured all our links were valid and publicly accessible, we noticed there is a reviewers-only link for Protocols.io. Please find the corrected link in the revised text.

Lines 646-652: The detailed protocol for coral cell dissociation is available on Protocols.io **(**https://www.protocols.io/private/1AB2BFF1ADF211F0AA040A58A9FEAC02**).** The immunoblot quantification data, R scripts, and the summary of experiments are available on Open Science Framework (https://doi.org/10.17605/OSF.IO/ZWP7G).

Reviewer 3:

The manuscript presents a well-developed and coherent description of the research problem, supported by appropriate and up-to-date references. The objective is clearly stated, and the study includes three different species of symbiotic cnidarians, which strengthens the comparative scope of the work. The results regarding HSP60 expression in the three species are interesting and contribute relevant information to the field. However, some of the figures do not meet a high standard of image quality (Figure 2 B), likely due to methodological issues related to the electrophoresis technique; in my view, this limitation compromise the validity of the results or their overall interpretation, but improving the image quality would enhance the clarity and impact of the presentation. If this is not possible, I suggest removing this species from the study or explaining more clearly how the low quality image and the normalization in the bar graph should be interpreted.

Reply: We thank the reviewer for this thoughtful and constructive assessment of our manuscript. We greatly appreciate the positive comments on the study design and on the comparative value of including three symbiotic cnidarian species. We also acknowledge and appreciate the concern raised regarding the image quality of Figure 2B.

We acknowledge that the quality of the representative blot image could be improved for clarity of presentation. However, **the interpretation of this result relies on normalized densitometric data generated from independent biological replicates rather than on qualitative assessment of the blot image alone**. As clarified in response to the related comments above, band intensities were quantified in ImageJ using defined regions of interest (ROIs), followed by local background subtraction and normalization. We have also

revised the manuscript to make the relationship between the representative blot image and the corresponding bar plot more explicit, including clarification that the plotted values reflect normalized densitometric measurements from independent biological replicates rather than repeated measurements from a single membrane image.

In response to concerns regarding exclusive reliance on β-actin normalization, we now include an additional analysis based on total-protein normalization in the Supplementary Information and have revised the Methods, Results, Discussion, and figure captions accordingly. This additional analysis improves the data interpretability for this species by introducing an alternative normalization framework that does not rely solely on the loading control.

For this reason, we have not removed the species from the analysis. Instead, we have revised the manuscript to clarify how the representative blot image relates to the normalized quantitative data. Retaining this species is important for the comparative design of the study, and the revised manuscript now presents the result with clarity and transparency.

Lines 169-173: While the representative blot image for *E. diaphana* (Figure 2B) is of lower visual quality, interpretation of this result is based on normalized densitometric measurements obtained from independent biological replicates rather than on visual assessment of the membrane image alone.

Although the methods section describes how the membrane signal was captured and quantified, the result associated with the membrane image itself is not addressed in the results section. It would be helpful to clarify how the quantification software identifies the area to be measured in relation to the figure showing the normalized intensity bar plots. Additionally, were the three replicates associated with the same membrane image, or does each bar correspond to an independent image/replicate.

Reply: We thank the reviewer for this helpful comment. We accept that the relationship between the representative membrane images and the corresponding densitometric quantification should be described more explicitly. In the revised manuscript, we have clarified both the image-analysis workflow and the definition of the replicates used for the normalized intensity plots.

Specifically, band intensity was quantified in ImageJ by defining a region of interest (ROI) around each band and subtracting the local background. The resulting HSP60 signal was then normalized to the corresponding **β-actin** signal and to the **total protein** for the complementary analysis included in the Supplementary Information. We have revised the Methods section and figure captions to make this workflow explicit.

We have also clarified that the membrane images shown in the figures are **representative blots**, whereas the normalized intensity plots summarize data obtained from **independent biological replicates**, not repeated measurements from a single membrane image. These revisions have now been incorporated into the manuscript to improve transparency and interpretability.

Lines 544-555: Band intensities were quantified using ImageJ/Fiji. Original chemiluminescent blot images were opened without contrast enhancement for

measurement. A uniform rectangular region of interest (ROI) was drawn around each band, and the same ROI size was maintained for all lanes on a given blot. Integrated density was measured for each target and loading-control band. To correct for the nonspecific signal, the background was measured from an adjacent membrane region lacking visible bands and subtracted from the raw band intensity. The corrected HSP60 signal for each lane was normalized to the corrected β-actin signal from the same lane. The resulting normalized values were used as relative densitometric measurements. Since a formal dilution-series experiment was not performed, the linear dynamic range of detection for HSP60 and β-actin was not explicitly validated here.

Lines 1029-1034: Representative western blots and corresponding densitometric quantification of HSP60 under control and heat-stress conditions. Densitometric measurements were performed in ImageJ after local background subtraction, and the HSP60 signal was normalized to the corresponding β-actin (B) and total protein signal (C). Data are presented as relative band intensity and represent the mean of technical replicates for each biological replicate (n=3, mean ± SE).

I suggest including the case of Hydra oligactis in the Introduction. Please review the following papers:

Bosch TC, Krylow SM, Bode HR, Steele RE. Thermotolerance and synthesis of heat shock proteins: these responses are present in Hydra attenuata but absent in Hydra oligactis. Proc Natl Acad Sci U S A. 1988 Nov;85(21):7927-31. doi: 10.1073/pnas.85.21.7927. PMID: 3186697; PMCID: PMC282326.

Clark MS, Fraser KP, Peck LS. Antarctic marine molluscs do have an HSP70 heat shock response. Cell Stress Chaperones. 2008 Spring:13(1):39-49. doi: 10.1007/s12192-008-0014-8. Epub 2008 Feb 7. PMID: 18347940; PMCID: PMC2666218.

Reply: We thank the reviewer for these recommendations. We agree that the case of *Hydra oligactis* is relevant to the broader context of variable heat-shock responses among cnidarians and provides a useful comparative example for the Introduction. We also appreciate the suggestion to consider the work of Clark et al. (2008). We have used this reference to further emphasize that heat-shock responses are not uniformly absent in environmentally specialized organisms, and that inducible HSP expression may persist even in taxa from stable thermal environments. This provides us with a useful comparative framework for interpreting lineage- and context-dependent variation in stress responses. We have therefore revised the Introduction to incorporate this literature in lines 108-115.

Lines 100-107: The differential induction of heat-shock response has also been reported in other cnidarians, such as the freshwater coelenterate *Hydra oligactis* (26-27). Researchers compared protein expression in this hydra species with that of *Hydra attenuata* and found that *H. oligactis* polyps are extremely sensitive to thermal stress, suggesting that the classically described heat shock response is absent in the system (26).While the heat shock response has been identified in mammalian systems and many aquatic species, it seems to be absent from some systems such as *Hydra oligactis* and *Euplotes focardii* (27)

May 22, 2026

RE: Life Science Alliance Manuscript #LSA-2025-03592-TR

Prof. Liza M. Roger
Arizona State University
School of Molecular Sciences
Walton Center For Planetary Health
777 E University Dr
TEMPE, Arizona 85281

Dear Dr. Roger,

Thank you for submitting your revised manuscript entitled "Conserved HSP60 structure with lineage- and context-specific regulation in cnidarians". We returned this work to Reviewers 2 and 3, who are now satisfied with no further concerns. would be happy to publish your paper in Life Science Alliance pending final revisions necessary to meet our formatting guidelines.

MANUSCRIPT ORGANIZATION AND FORMATTING:

To avoid unnecessary delays in the acceptance and publication of your paper, please read the following information carefully. Full guidelines are available on our Instructions for Authors page, https://www.life-science-alliance.org/authors

Submission of a paper that does not conform to Life Science Alliance guidelines will delay the acceptance of your manuscript.

-Please upload your main manuscript text as an editable doc file.
-Please upload your main and supplementary figures as single files.
-It is recommended to exclude figures from the manuscript text and leave them uploaded separately.
-Please add Keywords and a Category for your manuscript in our system.
-Please add the X and Bluesky handles of your host institute/organization, as well as your own, and/or one of the authors, in our system.
-The titles in both the system and the manuscript file must be consistent with each other.
-Please use the [10 author names et al.] format in your references (i.e., limit the author names to the first 10).
-Please add your main, supplementary figure, and table legends to the main manuscript text after the references section.
-please upload your Tables S6-S10 in editable .doc or excel format.
-Please add callouts for Figure 1A-C; 4A-C; 5A-C; S1; S2; S3a-B and table S10 to your main manuscript text.
-There appears to be a splice in the blot in Figure 2A, Beta-Actin row, after the first lane. Please include a clear line or separation between this lane and the remainder of the blot, and indicate in the figure legend if this blot was run on the same gel.

We encourage our authors to provide original source data, particularly uncropped/-processed electrophoretic blots and spreadsheets for the main figures of the manuscript. If you would like to add source data, we would welcome one PDF/Excel-file per figure for this information. These files will be linked as supplementary "Source Data" files.

It is Life Science Alliance policy that if requested, original data images must be made available to the editors. Failure to provide original images upon request will result in unavoidable delays in publication. Please ensure that you have access to all original data images prior to final submission.

If you are planning a press release on your work, please inform us immediately to allow informing our production team and scheduling a release date.

We welcome submissions of potential cover images for the issue of LSA in which your work would appear. If you have high quality images associated with this work, please feel free to email these, with a caption, to the journal office.

LSA encourages authors to provide a 30-60 second video where the study is briefly explained. These videos will be appear embedded with the manuscript online at Life Science Alliance, and on social media to promote the published paper and authors (for examples, see https://docs.google.com/document/d/1-UWCfbE4pGcDdcgzcmiuJl2XMBJnxKYeqRvLLrLSo8s/edit?usp=sharing). Corresponding or first-authors are welcome to submit the video. Please submit only one video per manuscript. The video can be emailed to contact@life-science-alliance.org

FINAL FILES:

To upload the final version of your manuscript, please log in to your account: https://lsa.msubmit.net/cgi-bin/main.plex
You will be guided to complete the submission of your revised manuscript and to fill in all necessary information. Please get in touch in case you do not know or remember your login name.

The following items are required for acceptance.

-- An editable version of the final text (.DOC or .DOCX) is needed for copyediting (no PDFs).

-- High-resolution figure, supplementary figure and video files uploaded as individual files: See our detailed guidelines for preparing your production-ready images, https://www.life-science-alliance.org/authors

-- Summary blurb (enter in submission system): A short text summarizing in a single sentence the study (max. 200 characters including spaces). This text is used in conjunction with the titles of papers, hence should be informative and complementary to the title. It should describe the context and significance of the findings for a general readership; it should be written in the present tense and refer to the work in the third person. Author names should not be mentioned.

The license to publish form must be signed before your manuscript can be sent to production. A link to the license to publish form will be available to the corresponding author only. Please take a moment to check your funder requirements.

Reviews, decision letters, and point-by-point responses associated with peer-review at Life Science Alliance will be published, alongside the manuscript. If you do want to opt out of having the reviewer reports and your point-by-point responses displayed, please let us know immediately.

Thank you for your attention to these final processing requirements. Please revise and format the manuscript and upload materials as soon as you are able.

Thank you for this interesting contribution to the literature. We look forward to publishing your paper in Life Science Alliance.

Sincerely,

Tim Fessenden
Scientific Editor
Life Science Alliance

Reviewer #2 (Comments to the Authors (Required)):

I recommend this manuscript is acceptable for publication in present version.

Reviewer #3 (Comments to the Authors (Required)):

The manuscript has been substantially improved following the peer review process, with the authors providing clear and thorough responses to the reviewers' comments. In particular, the statistical methods are now properly described, and the calculation of protein expression levels by image analysis of the Western blot has been clarified (lines 169 and 173 Figure 2B and C). However, the membrane images for HSP60 and actin exhibit noticeable technical issues. These may be attributed to factors such as suboptimal running buffer composition, electrophoresis voltage conditions, or transfer efficiency. Despite these limitations, the decision to include these images is commendable, as it reflects transparency and scientific integrity, especially in a context where image manipulation has been a concern in the literature.

June 16, 2026

RE: Life Science Alliance Manuscript #LSA-2025-03592-TRR

Prof. Liza M. Roger
Arizona State University
School of Molecular Sciences
Walton Center For Planetary Health
777 E University Dr
TEMPE, Arizona 85281

Dear Dr. Roger,

Thank you for submitting your Research Article entitled "Conserved HSP60 structure with lineage- and context-specific regulation in cnidarians". It is a pleasure to let you know that your manuscript is now accepted for publication in Life Science Alliance. Congratulations on this interesting work.

The final published version of your manuscript will be deposited by us to PubMed Central upon publication.

Your manuscript will now progress through copyediting and proofing. It is journal policy that authors provide original data upon request.

Your article will publish open access upon publication under a CC-BY license.

Reviews, decision letters, and point-by-point responses associated with peer-review at Life Science Alliance will be published, alongside the manuscript. If you do want to opt out of having the reviewer reports and your point-by-point responses displayed, please let us know immediately.

IMPORTANT: If you will be unreachable at any time, please provide us with the email address of an alternate author. Failure to respond to routine queries may lead to unavoidable delays in publication.

Scheduling details will be available from our production department. You will receive proofs shortly before the publication date. Only essential corrections can be made at the proof stage so if there are any minor final changes you wish to make to the manuscript, please let the journal office know now.

DISTRIBUTION OF MATERIALS:
Authors are required to distribute freely any materials used in experiments published in Life Science Alliance. Authors are encouraged to deposit materials used in their studies to the appropriate repositories for distribution to researchers.

You can contact the journal office with any questions, contact@life-science-alliance.org

Again, congratulations on a very nice paper. I hope you found the review process to be constructive and are pleased with how the manuscript was handled editorially. We look forward to future exciting submissions from your lab.

Sincerely,

Tim Fessenden
Scientific Editor
Life Science Alliance
